# A Potential Role for CHH DNA Methylation in Cotton Fiber Growth Patterns

**DOI:** 10.1371/journal.pone.0060547

**Published:** 2013-04-12

**Authors:** Xiang Jin, Yu Pang, Fangxing Jia, Guanghui Xiao, Qin Li, Yuxian Zhu

**Affiliations:** 1 State Key Laboratory of Protein and Plant Gene Research, College of Life Sciences, Peking University, Beijing, China; 2 National Center for Plant Gene Research (Beijing), Beijing, China; Nanjing Agricultural University, China

## Abstract

DNA methylation controls many aspects of plant growth and development. Here, we report a novel annual growth potential change that may correlate with changes in levels of the major DNA demethylases and methyltransferases in cotton ovules harvested at different times of the year. The abundances of DNA demethylases, at both the mRNA and protein levels, increased significantly from February to August and decreased during the remainder of the 12-month period, with the opposite pattern observed for DNA methyltransferases. Over the course of one year, substantial changes in methylcytosine content was observed at certain CHH sites (H = A, C, or T) in the promoter regions of the *ETHYLENE RESPONSIVE FACTOR 6* (*ERF6*), *SUPPRESSION OF RVS 161 DELTA 4* (*SUR4*) and *3-KETOACYL-COA SYNTHASE 13* (*KCS13*), which regulate cotton fiber growth. Three independent techniques were used to confirm the annual fluctuations in DNA methylation. Furthermore, in homozygous RNAi lines specifically targeting REPRESSOR OF SILENCING 1 (ROS1, a conserved DNA demethylase domain), promotion of DNA methylation significantly reduced fiber growth during August.

## Introduction

Most living organisms maintain an internal clock to estimate the passage of time and schedule physiological processes using a circadian clock of approximately 24 hours [1], [2]. This short-term time-keeping mechanism is precisely regulated by a network of transcriptional and posttranscriptional events [3], [4], most of which involve the remodeling of chromatin through changes in histone acetylation [5]–[7]. Vertebrates have two additional types of long-term time-keeping mechanisms that enable animals to track, anticipate, and prepare for seasonal changes [8], [9]. Whereas one measures an interval of several months, the other oscillates with a periodicity of approximately one year. In certain plants, increased dimethylation of lysine residue 9 and trimethylation of lysine 27 on histone H3 associated with the 5′ regions of the *FLOWERING LOCUS C* (*FLC*) gene during the winter season may serve as an epigenetic memory system that represses *FLC* transcription and initiates flowering at the end of winter [9], [10]. This “histone code”, which specifies a silent chromatin state, appears to be conserved between animals and plants [5], [6], [10].

Our previous studies of fiber development in cotton (*Gossypium hirsutum* L.) revealed a striking variation in the growth of ovules that were harvested from plants grown in fully automated walk-in growth rooms and immediately cultured for 6 d [11]–[13]. We observed a marked variation in fiber length (1.4±0.1 mm *vs.* 3.8±0.3 mm for ovules harvested in Feb to March *vs*. May to June, respectively) that seemed attributable only to a difference in the time of the year because all other parameters, including photoperiod, temperature, moisture, and the physiological conditions of the harvested ovules, were identical.

To elucidate the molecular mechanisms that control this phenomenon, we systematically measured fiber lengths of cultured ovules collected at different times of the year over a 3-year period. We also constructed a large cDNA microarray containing all universal expressed sequence tags (UniESTs) obtained from sequencing of the upland cotton transcriptome, and used this to profile mRNA samples prepared from ovules harvested at different times of the year.

## Materials and Methods

### Plant Materials

Cotton plants (*Gossypium hirsutum* cv. Xuzhou 142) were grown in a soil mixture [11] in fully automated walk-in growth rooms with 300 µmol/m^2^/s average light intensity, 60% relative humidity, and temperatures set to 30°C during the light period and 28°C during the dark (12-h light/dark cycle). These conditions were consistently maintained throughout the year. For bisulfite sequencing or Southern blotting experiments, the 2^nd^ to 5^th^ cotton bolls on the 2^nd^ to 5^th^ fruiting branches were harvested during 11 a.m. to 2 p.m. to prepare 7-dpa (day post anthesis) samples. Bolls from at least 10 flowering plants were mixed for each ovule culture experiment to minimize differences in growth potential. For every one to two years, we obtained new batches of field-harvested cotton seeds from the genetic stock maintained by Chinese Cotton Research Institute.

### RNA Extraction and qRT-PCR

Cotton ovules harvested at the specified times were frozen in liquid nitrogen before being ground to a fine powder with a mortar and pestle using a modified hot borate method [11]. Total RNA was extracted from cotton ovules as described [11], and cDNA was reverse-transcribed from 5 µg total RNA. RNA samples representing the four seasons were prepared from ovules harvested during 11 a.m. to 2 p.m. to avoid any possible influence of circadian clock on the samples, and were mixed from a large number of cotton plants (>30) to minimize individual variation. All samples were collected within the first 16 d of the specified month. A panel of 20 housekeeping genes (Table S5) was used for internal controls in all qRT-PCR experiments. All qRT-PCR analyses were performed using three independent RNA samples prepared from at least three cotton plants as biological replicates and three equal reaction systems for each RNA sample were used as technical replicates.

### Construction and Hybridization of cDNA Microarrays

The cDNA microarray was constructed using cotton ESTs with GenBank accession numbers DR452281–DR463972, EV481736–EV499360, and ES790335–ES852447 by CapitalBio Corp, essentially as described [11]. Briefly, 28,178 cotton cDNAs and 8 intergenic yeast sequences (used as external controls) were amplified and printed onto amino silane slides. Fluorescent dye-labeled DNA (with swap-dye experiments performed on each of the four independent RNA samples as a technical replicate) was produced using Eberwine’s linear RNA amplification method [11]. We used a common reference that consisted of equal amounts of RNA prepared from the four different seasons to minimize background noise in each of the hybridization experiments. All common references were arbitrarily assigned a value of 1 throughout the experiment. Microarray spots with false discovery rate-corrected P values <0.001 (by an F-test that tested the existence of a possible seasonal effect for certain gene expressions) were regarded as differentially expressed genes. Hierarchical clustering with the average linkage method was employed only on those genes that showed significant preferential expression in one or more seasons, and cluster data were visualized using the Treeview program [14].

### Preparation of Antibodies and Western Blotting

We used Peptide-Antigen Finder software (China Peptide Corp.) to design four oligopeptides (MDQNGSGGDADNFDW and CNGSNYNKRRNLGYDL from DRM1/2, TEGKPGRPRKPATPK and THRRQNTHPQKLSNR from ROS1, Figure S2A) for immunizing rabbits to produce specific polyclonal antibodies. Before use for western blotting, the antibodies were purified using the respective oligopeptide as the affinity column tag. Total cytosolic proteins from cotton ovules at different times of the year were extracted [15] and loaded at 20 µg per lane for the blotting. A commercial antibody against an *Arabidopsis* UBQ (1∶1,000 anti-ubiquitin, Abcam ab7254, Santa Cruz, CA) was used as a loading control.

### Bisulfite Sequencing

Bisulfite sequencing was performed using bisulfite-treated genomic DNA isolated from cotton ovules at different times of the year, as previously described [16], [17]. The upstream regions of *SUR4* (*SUPPRESSION OF RVS 161 DELTA 4*), *KCS13* (*3-KETOACYL-COA SYNTHASE 13*), *ERF6* (*ETHYLENE RESPONSIVE FACTOR 6*), and *TUB3* (*β-tubulin 3*), as well as the coding region of *eIF2A* (*eukaryotic translation initiation factor 2A*) were sequenced using DNA samples prepared from February 2010 to February 2011. Sequencing was performed on 17 unique “non-sister” individual bisulfite clones obtained from independent PCR reactions using primers reported in Table S6. Sequencing was performed by a commercial service (Invitrogen Corporation, Shanghai).

### Methylation-sensitive Endonuclease Digestion

Genomic DNA was isolated from 7-dpa cotton ovules harvested at different times of the year. Aliquots (1 µg) were digested by incubation with 1 µl *Bsl*I (10,000 U/ml) in NEBuffer 3 at 55°C for 16 h, with 1 µl *HinF*I (10,000 U/ml) in NEBuffer 4 at 37°C for 16 h, or with 1 µl *BstX*I (10,000 U/ml) in NEBuffer 3 at 37°C for 16 h. One twentieth of the digested DNA sample was used as the template for each PCR analysis.

### Southern Blots

To detect cleavage of methylation-sensitive sites, 20 µg genomic DNA was digested thoroughly (using 20 times the amount of enzyme specified in the previous section using methylation-sensitive *Bsl*I, *HinF*I, or *BstX*I, loaded onto electrophoretic gels, and blotted prior to hybridization and detection using the DIG High Prime DNA Labeling and Detection Starter Kit II (Roche) with probe sequences specified in each respective figure panel. Signal ratios from each experiment were calculated by dividing the intensity measured for full-length DNA by that of the methylation-sensitive endonuclease-cleaved fragment.

### Production of Transgenic Cotton Plants Expressing RNAi Vectors Targeted to a Conserved Domain of the *ROS1* mRNA

The 549-nucleotide (71–619 bp in the ROS1 CDS) coding region that is most highly conserved in *ROS1* homologs was amplified using the primers specified in Table S6 as described previously [18]. The PCR fragment was first cloned into the pBlueScript SK vector with a 1-kb spacer ligated between the *Eco*RV and *Hin*dIII sites. The sense fragments of *ROS1* were digested using *Sac*I and *Sma*I, and the antisense fragments were digested using *Xho*I and *Sal*I. The sense, antisense, and spacer sequences were then cloned into pCAMBIA2300 using the *Kpn*I and *Sac*I restriction sites, placing their expression under the control of the fiber-specific E6 promoter. The resulting construct was transformed into CCRI 24, a Chinese cotton variety, using the *Agrobacterium*-mediated method. Genetic segregation tests, genomic DNA PCR, and Southern blotting were used to confirm that the transgenic plants contained a single T-DNA insertion.

### Statistical Analysis

Whenever applicable, data were evaluated by one-way ANOVA combined with Tukey’s test to obtain statistical significance values. Throughout this work: *p<0.05; **p<0.01; ***p<0.001.

## Results

### An Annual Growth Potential Change Effects Cotton Plant, Especially Fiber Development

**Figure 1:**
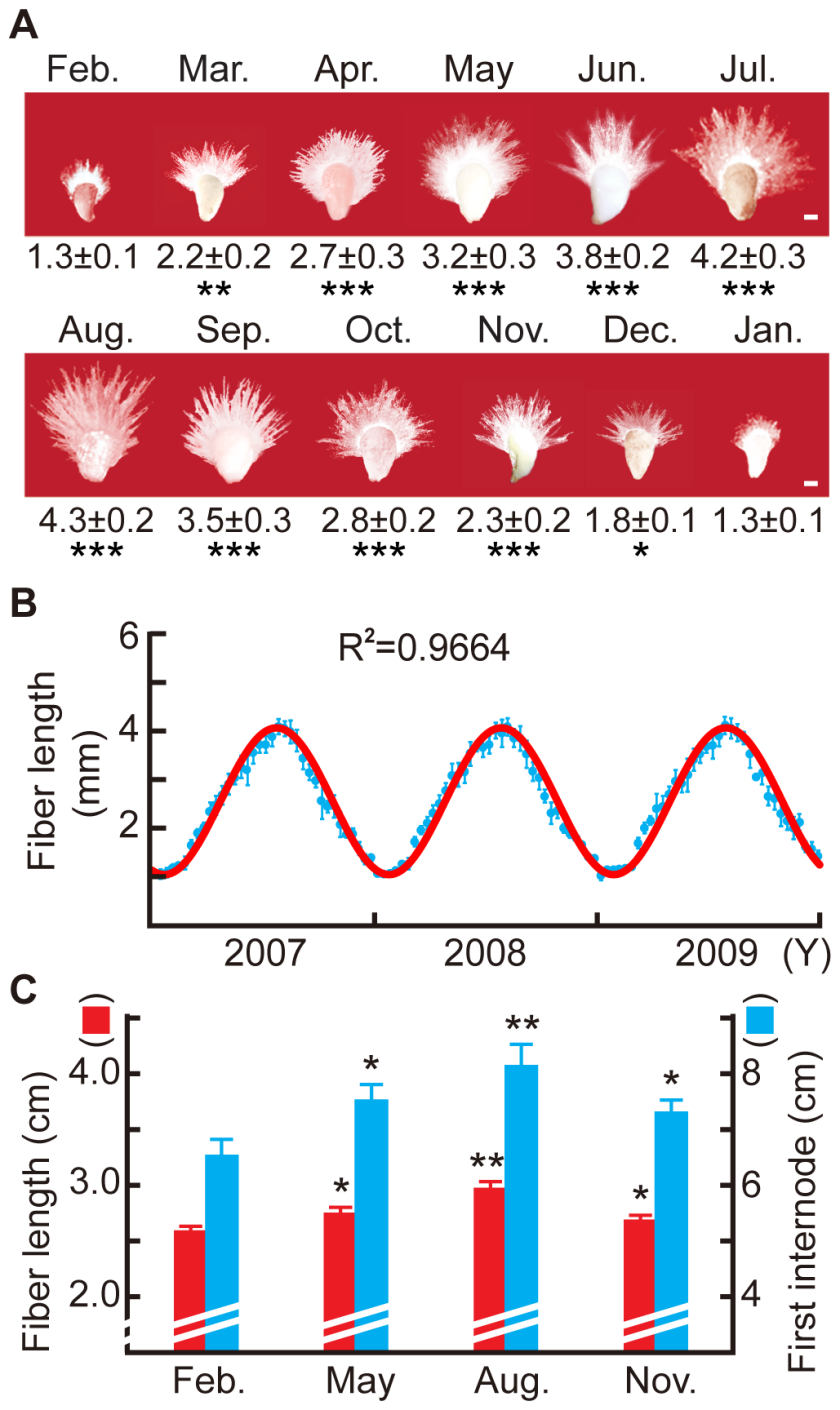
Annual growth potential change of cotton plants. (A) Cotton ovule growth potential as a function of month in which ovules were harvested. Ovules were harvested 1 dpa during the month indicated, cultured for 6 d, and measured for fiber length. Numbers indicate fiber length (mean ± SE, in mm). Each ovule in this panel is a representative of thirty in the same culture. (B) Growth of cotton fibers from ovules harvested over the same monthly cycle for three consecutive years. (C) Cotton fiber length and first main stem internode length *in planta* in four different seasons. Error bars, SE. In (A–C), n = 6; In (A,C), *p<0.05, **p<0.01, ***p<0.001.

Experiments involving cultured ovules were conducted over three consecutive years from 2007 to 2009. Ovules (1 day post-anthesis [dpa]) were harvested from cotton plants and cultured for 6 d before images were acquired and fiber lengths recorded (Figure 1A). Plants that flowered in January and February showed minimal growth (fiber cells elongated to 1.3±0.1 mm), whereas those that flowered during August showed maximal growth (4.3±0.2 mm). A similar growth pattern, albeit at a lower magnitude, was observed with regard to ovule size (Figure 1A). The same growth potential change was observed each year, with an R^2^ of 0.9664 (Figure 1B). When first internodes of main stem and cotton fibers that had matured *in planta* in walk-in growth rooms were measured, we recorded a similar growth pattern change which indicates the changes in growth rates were similar, albeit significantly smaller (Figure 1C).

### Genes Involved in Multiple Biological Processes are Differentially Expressed Over the Course of a Year

A cotton ovule cDNA microarray with 28,178 UniESTs (GEO accession no. GPL8569) was prepared and probed with RNA samples obtained from 7-dpa ovules harvested at four different time points (May, August, November, and February; Table S1). This identified 202 genes that were significantly upregulated in August and 33 that were upregulated in February (Table S2). Further analysis using GOEAST (http://omicslab.genetics.ac.cn/GOEAST/; [19]) revealed 14 biological process categories that were significantly enriched at some time over the course of a year (Figure S1), with DNA methylation showing the lowest P value (corrected for false discovery rate; Table S3).

### DNA Methylation may be Responsible for the Annual Growth Potential Change in Cotton

In higher plants, DOMAINS REARRANGED METHYLASE 1/2 (DRM1/2) is responsible for *de novo* methylation in all sequence contexts, including CG, CHG, and asymmetric CHH sites [20], [21], where H is any nucleotide but guanine. The plant-specific CHROMOMETHYLASE 3 (CMT3) acts redundantly with DRM1/2 to maintain CHG and CHH methylation in a locus-specific manner [20], [22]–[24]. DICER-LIKE 3 (DCL3) is required for biogenesis of the 24-nt siRNAs required for sequence targeting during RNA-directed DNA methylation [25], [26]. In *Arabidopsis*, DNA is demethylated by the DNA glycosylation activities of ROS1 and DEMETER (DME) [26], [27]. We obtained putative full-length cDNAs for all five genes identified in GO: 0006306, including DCL3, two DNA methylases (DRM1/2 and CMT3), and two DNA demethylases (ROS1 and DME; Table S4).

**Figure 2:**
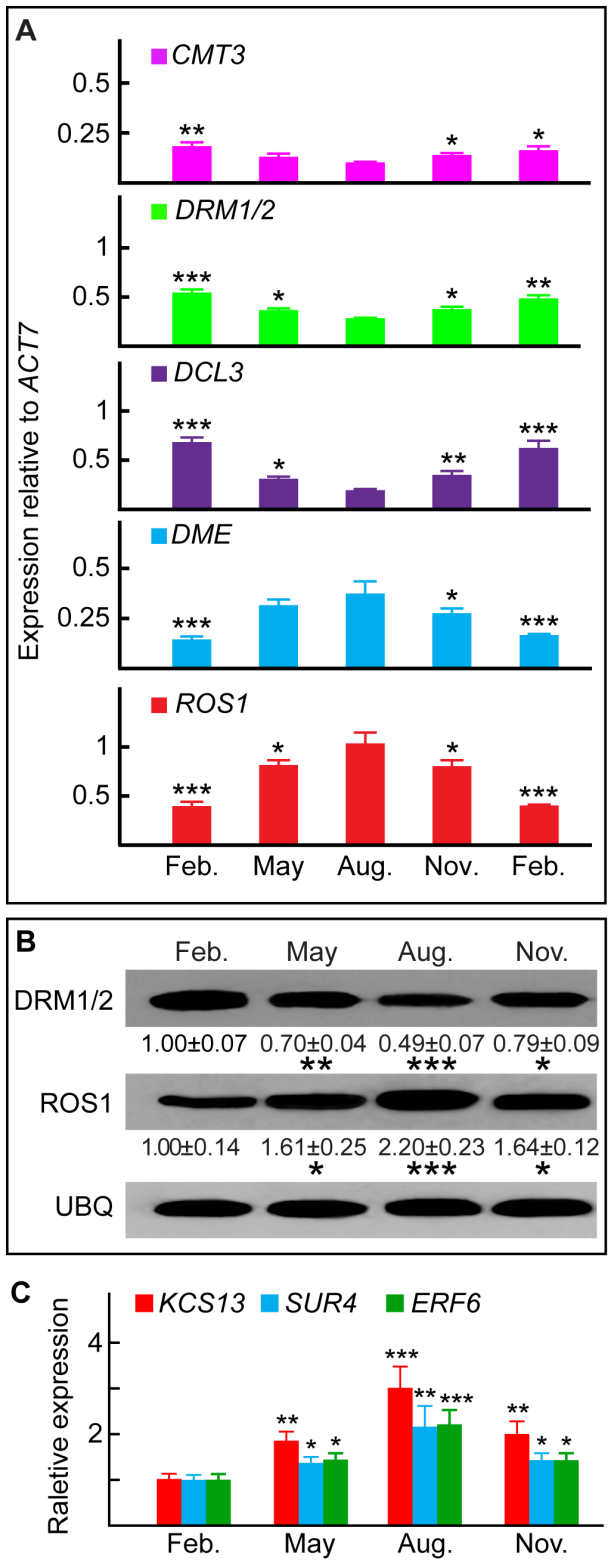
Annual changes in DNA methylation patterns in cotton ovules. (A) qRT-PCR analysis of DNA methylation and demethylation genes reported in GO: 0006306. The *ACT7* transcript, normalized against that of all 20 housekeeping genes reported in Table S5, was used as the internal standard. (B) Western blot analysis of DRM1/2 and ROS1. May, August, and November signal intensities were normalized to those from February (arbitrarily set to 1). Values (mean ± SE from three independent experiments) are shown beneath representative bands. Cotton UBQ was used as a loading control. (C) *KCS13*, *SUR4* and *ERF6* transcript levels changed over the course of the year, as quantified by qRT-PCR. May, August, and November values were normalized to February values (arbitrarily set to 1).

Quantitative real-time PCR (qRT-PCR) was next performed on RNA samples prepared from tissues harvested at different times of the year. As expected, the expression of 20 selected housekeeping genes remained unaltered over time (Table S5, and see Table S6 for primers). In contrast, there was a substantial variation in expression of genes encoding DNA methylation and demethylation functions. Consistent with data obtained using microarray analysis (Table S2), the highest levels of *CMT3, DRM1/2*, and *DCL3* transcripts were observed in February, followed by a gradual and significant decrease from May to August and a similar increase from November to February (Figure 2A, top three panels). By contrast, *ROS1* and *DME* transcript levels increased steadily from February to August and decreased from November to February (Figure 2A, bottom two panels). Western blotting with antibodies specific for cotton DRM1/2 and ROS1 (see Figure S2 for production and specificity of antibodies used) showed similar changes in the levels of both proteins over the 1-year period (Figure 2B).

Transcript levels for three genes important for fiber growth, *ERF6*, *SUR4,* and *KCS13* [11]–[13], were significantly higher in ovules harvested during August than in ovules harvested during February, with intermediate levels in ovules harvested during May and November (Figure 2C).

### Annual Changes in CHH DNA Methylation are Observed in the Promoter Regions of Several Important Growth Regulatory Genes

**Figure 3:**
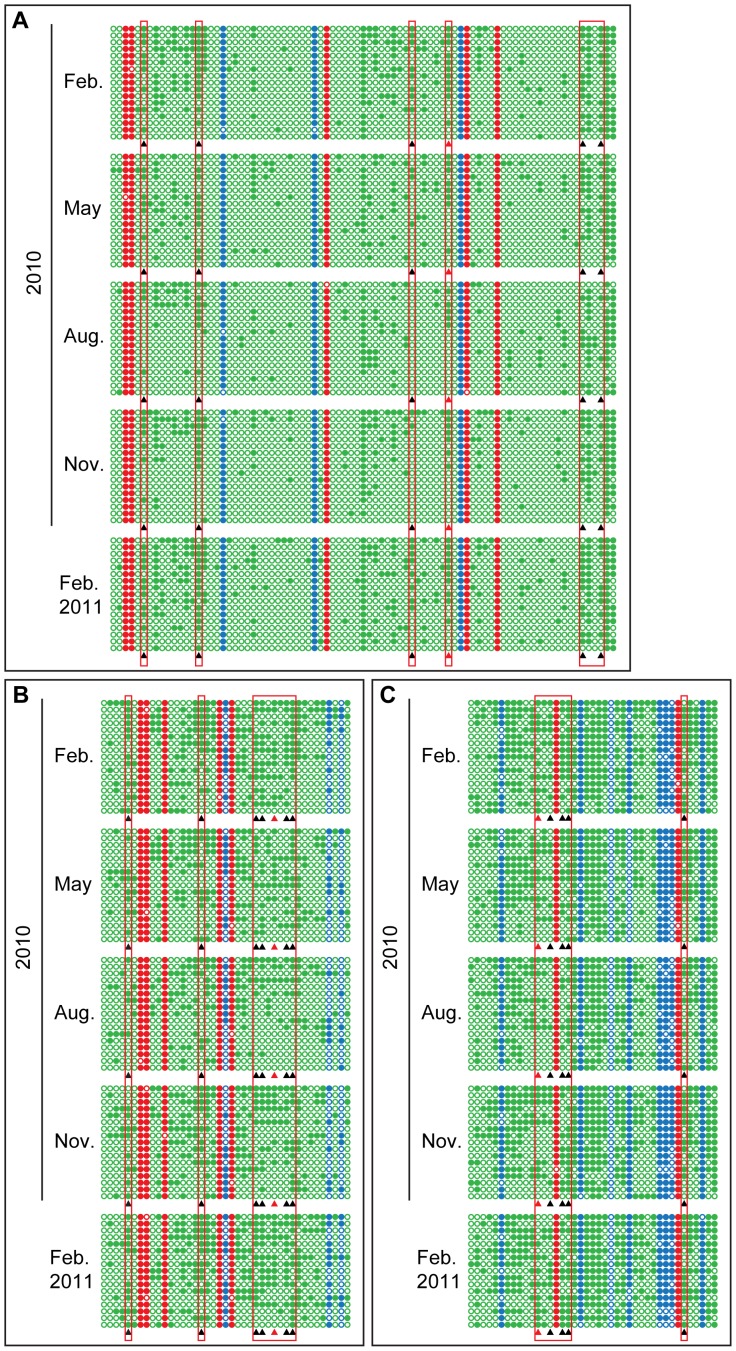
Bisulfite sequencing of *ERF6*, *SUR4*, and *KCS13* upstream regions over one year. The fragments to be examined were amplified by sequence-specific PCR primers after treating the template DNA with bisulfite. 17 unique “non-sister” individual clones from independent PCR reactions were selected for sequencing. Each line represents one unique “non-sister” individual bisulfite sequencing result. Only cytosines are shown using red for CG context, blue for CHG, and green for CHH. Open circles, unmethylated cytosines; closed circles, methylated cytosines; black triangles, cytosines showed annual methylation changes; red triangles, cytosine sites used for methylation-sensitive endonuclease digested PCR and Southern assay. The same designations were used for all bisulfite sequencing data reported in the current work. (A) The sequence from 272 to 662 nt in DQ464372 from *ERF6* upstream region was analyzed for DNA methylation. (B) The sequence from 322 to 646 nt in JQ922563 from *SUR4* upstream region was analyzed for DNA methylation. (C) The sequence from 1641 to 1939 nt in JQ922562 from *KCS13* upstream region was analyzed for DNA methylation.

The upstream regions of *ERF6*, *SUR4* and *KCS13* were characterized by bisulfite sequencing [16], [17] to determine whether DNA methylation patterns change over the course of a year and whether the same set of cytosine residues are reversibly methylated. Site-specific patterns of cytosine methylation and demethylation were observed in cotton materials harvested from February 2010 to February 2011. Comparison of samples from February and August identified six CHH sites in the region of *ERF6* upstream region that had a >2-fold difference in methylation, with intermediate methylation levels in May and November (Figure 3A, see Table S7 for statistics). Almost identical annual changes in methylation patterns were observed in seven and six CHH sites in the upstream regions of *SUR4* and *KCS13*, respectively (Figure 3B,C). These annual variations in methylation occurred only at CHH sites, with little change at CG or CHG sites in the upstream regulatory regions of any of the three genes (Figure 3).

Two housekeeping genes selected from Table S5 were used as control sequences to ensure the accuracy of the bisulfite sequencing data. In the predicted coding region of the *EUKARYOTIC TRANSLATION INITIATION FACTOR 2* (*eIF2A*) gene, there is very low rates of DNA methylation throughout the year (Figure S3A). When the CG- and CHG-rich upstream region of *TUBULIN 3* (*TUB3*) was used, we observed maximal but constant methylation in bisulfite sequencing (Figure S3B).

**Figure 4:**
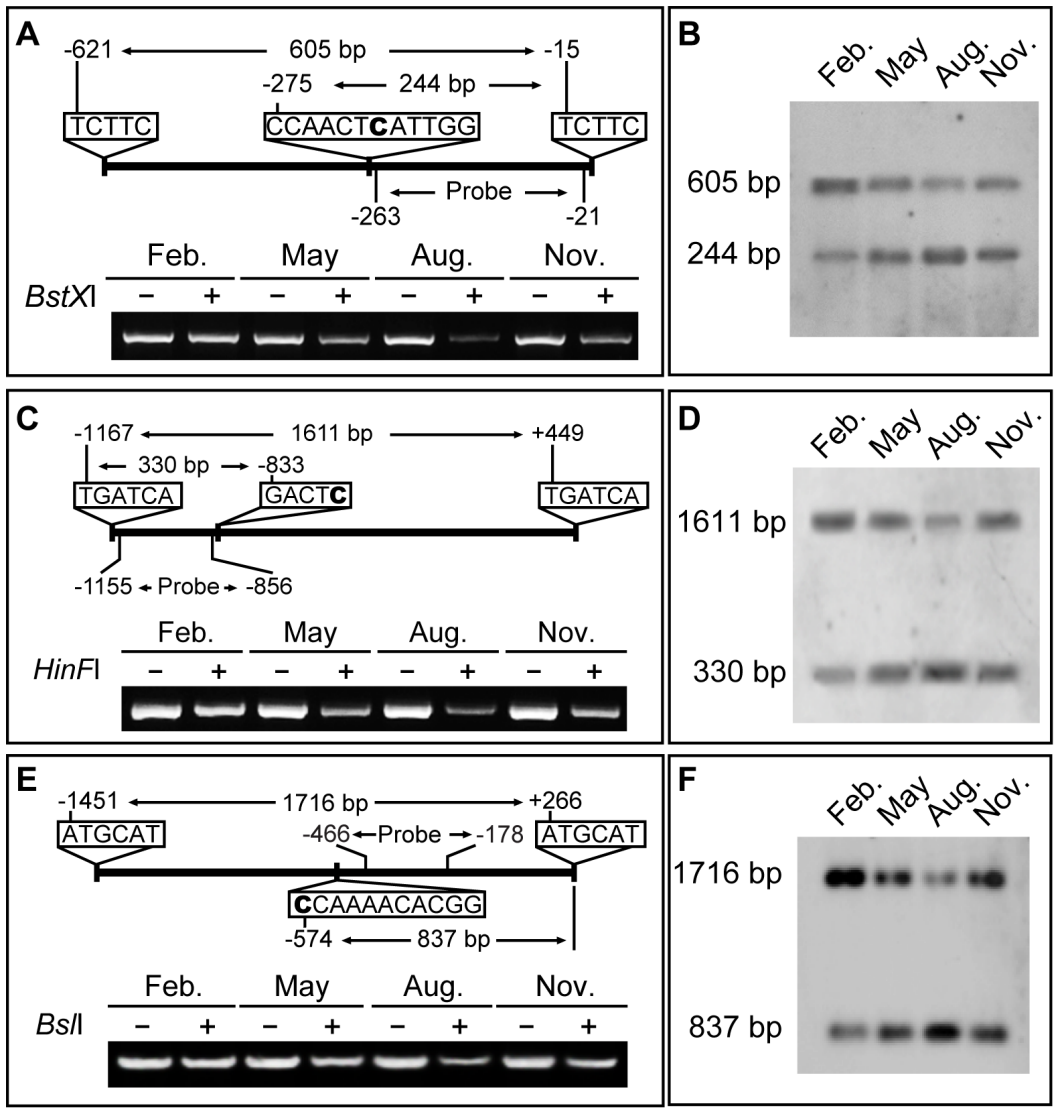
Methylation-sensitive endonuclease digested PCR and Southern analysis of *ERF6*, *SUR4*, and *KCS13* upstream regions over one year. (A) Methylation-sensitive endonuclease digested PCR amplification of *ERF6* upstream region. Top: schematic diagram of the identification of a methylation-sensitive *BstX*I digenstion site (CCANNNNNNTGG) at −275 bp of the *ERF6* promoter. The bold C indicates a CHH site with annual methylation pattern change, corresponding to the cytosine labelled with red triangles in Figure 3A. Bottom: PCR amplification using genomic DNA with (+) or without (−) *BstX*I digestion. (B) Southern blot of genomic DNA harvested at different times of the year, first digested by a methylation non-sensitive endonuclease *Mbo*II (TCTTC) to obtain a full length fragment of 605 bp from −621 to −15 of *ERF6* upstream regions, then digested thoroughly with *BstX*I, and probed with the fragment from −263 to −21 nt. The signal intensities of the band of *BstX*I-cleaved 244 bp changed at different time-of-year (see Table S8), indicating the methlytion levels of this CHH site were different, consistent with the bisulfite sequencing data in Figure 3A and methylation-sensitive endonuclease digested PCR results in Figure 4A. The same methylation-sensitive endonuclease digested PCR experiments were performed for the upstream regions of *SUR4* (C) and *KCS13* (E), except the methylation-sensitive endonucleases used were *HinF*I and *Bsl*I, respectively. Further, the same methylation-sensitive endonuclease digested Southern experiments were performed for the upstream regions of *SUR4* (D) and *KCS13* (F), except the genomic DNA were first digested by *Bcl*I (TGATCA) and *NSi*I (ATGCAT), then digested by methylation-sensitive endonucleases *HinF*I (GANTC) and *Bsl*I (CCNNNNNNNGG), respectively. The signal intensities of *HinF*I- and *Bsl*I-cleaved 330 bp and 837 bp changed similarly (see Table S8).

Two additional approaches were employed to confirm the annual fluctuations in cytosine methylation pattern. First, the methylation-sensitive endonucleases *BstX*I, *HinF*I and *Bsl*I and were used to digest the upstream regions of *ERF6*, *SUR4* and *KCS13*, respectively. The schematic diagrams of the methylation-sensitive edonuclease digestion assay are illustrated in the upper panels of Figure 4A, C and E. For all three genes, the greatest cleavage (and thus lowest amplification) was observed in August (lower panels of Figure 4A,C,E), which is consistent with the observed decrease in methylation in August. From November to February, there was a steady increase in amplification, indicating decreased cleavage, consistent with an increase in methylation.

Next, Southern blot analysis of *BstX*I-cleaved genomic DNA using a probe from the *ERF6* upstream region revealed substantially more intense hybridization signals corresponding to the 244-bp fragment (representing the fraction of unmethylated CHH at the site shown by a red triangle in Figure 3A) in August, whereas the signal was more intense for the 605-bp fragment (representing undigested and thus methylated CHH site) in February (Figure 4B). Based on this analysis, samples from May and November yielded methylated/unmethylated ratios close to 1, whereas the ratios for August and February samples were 0.4–0.5 and ∼2, respectively (Table S8). These measured ratios are consistent with the observed changes in CHH methylation. Similar patterns were observed using probes derived from upstream regions of *SUR4* and *KCS13* and DNA digested with *HinF*I and *Bsl*I, respectively (Figure 4D,F; see Table S8 for statistics).

### CHH DNA Methylation is Associated with Fiber Growth Potential in Several ^ROS1^RNAi Cotton Lines

**Figure 5:**
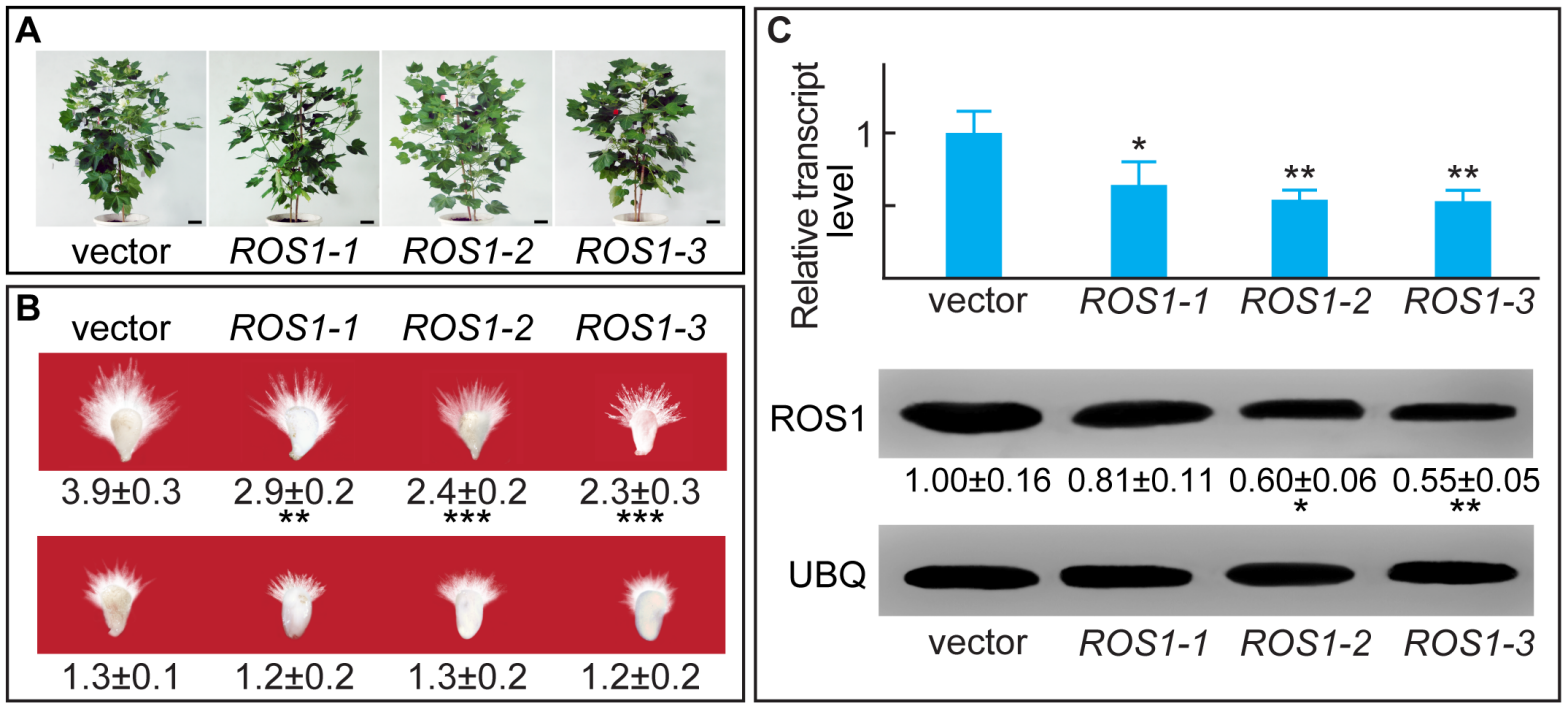
Phenotype and genetic identification of ^ROS1^RNAi lines. (A) Homozygous ^ROS1^RNAi cotton lines at flowering. Vector plants carry the empty vector and showed identical properties with the parent. (B) Cotton ovules from RNAi lines that flowered during August (upper panel) or February (lower panel) were cultured for 6 d before being photographed for fiber measurement. (C) Analysis of *ROS1* transcripts in ovules from various ^ROS1^RNAi lines by qRT-PCR (upper panel) and western blotting (lower panel).

**Figure 6:**
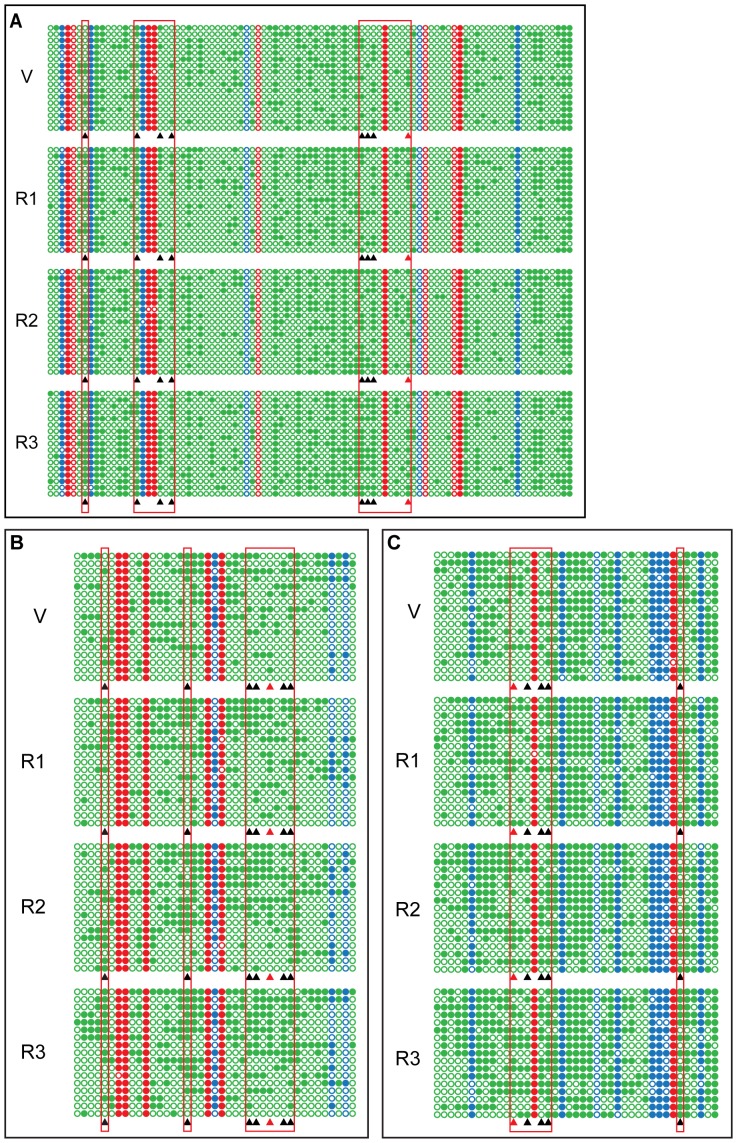
Bisulfite sequencing of *ERF6*, *SUR4*, and *KCS13* upstream regions in ^ROS1^RNAi lines. The same primers were used for bisulfite treated PCR and sequencing as in Figure 3. (A), promoter region of *ERF6*; (B), promoter region of *SUR4*; (C), promoter region of *KCS13*; V, RNAi line with empty vector; R1–R3, RNAi line *ROS1-1* to *ROS1-3*. All symbols are same to Figure 3.

**Figure 7:**
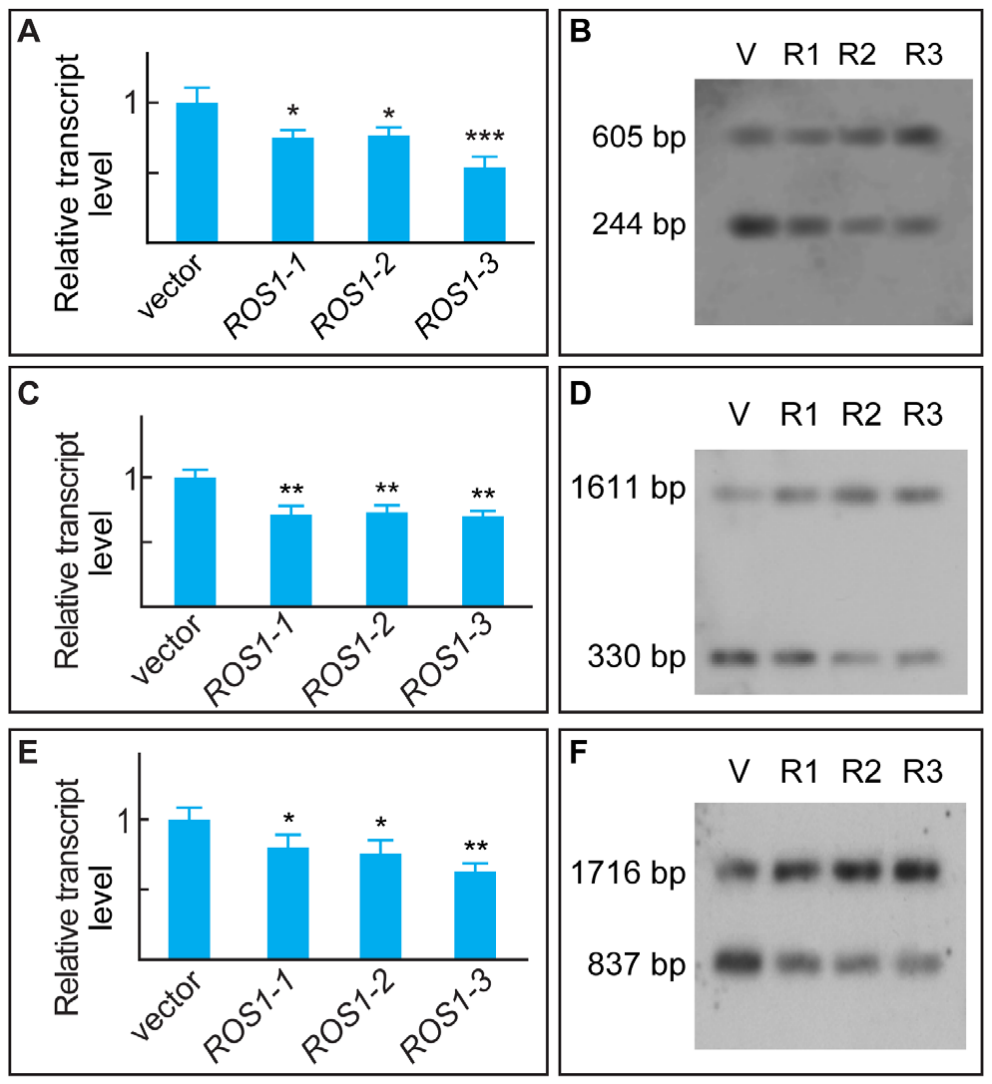
Methylation-sensitive endonuclease digested PCR and Southern analysis of *ERF6*, *SUR4*, and *KCS13* upstream regions in ^ROS1^RNAi lines. (A) Analysis of relative *ERF6* transcription in ovules from ^ROS1^RNAi lines by qRT-PCR. The level of *ERF6* transcripts in ovules from the empty vector line (V) was arbitrarily defined as 1. (B) Southern blot analysis of genomic DNA prepared from ^ROS1^RNAi lines digested thoroughly with *BstX*I. See detailed information in Figure 4 legend. Similar qRT-PCR experiments were performed for *SUR4* (C) and *KCS13* (E) transcriptions, as well as similar Southern experiments for *SUR4* (D) and *KCS13* (F), respectively. Note the reduced intensities of the *BstX*I-, *HinF*I- and *Bsl*I-cleaved bands in all three RNAi lines compared to the vector line.

Several homozygous *ROS1* RNA interference (^ROS1^RNAi) lines [18] were generated to examine whether the rate of DNA methylation reflects the potential for fiber growth *in vivo* (Figure 5A). Ovules of ^ROS1^RNAi plants that flowered during August produced fiber lengths similar to those from plants that flowered during November or December (Figure 5B), suggesting that perturbed DNA methylation inhibited fiber growth. All three lines contained significantly lower levels of *ROS1* transcripts (Figure 5C, upper panel) and ROS1 protein (Figure 5C, lower panel). Bisulfite sequencing data were consistent with these trends, showing November- or December-like CHH methylation in various ^ROS1^RNAi lines, especially in *ROS1-2* and *ROS1-3* (Figure 6). Transcription of *SUR4*, *KCS13* and *ERF6* was inhibited significantly in all three ^ROS1^RNAi lines, as confirmed by qRT-PCR (Figure 7A,C,E). Compared with the vector line, the band intensities for the *BstX*I-cleaved 244-bp unmethylated fragment from the upstream region of *ERF6* were significantly lower in all three RNAi lines, as verified by Southern blotting (Figure 7B). Similar results were obtained for the upstream regions of *SUR4* and *KCS13* after digestion with *HinF*I and *Bsl*I, respectively (Figure 7D,F).

## Discussion

It is remarkable to find out that flowering plants keep track of the time of year by reducing or promoting DNA methylation mainly at CHH sites. Epigenetic modifications of chromatin at the DNA or histone level are considered to be one of the major forces that influence gene expression [26], [28]. Several groups have correlated genome-wide changes in methylation patterns with physiological and developmental responses. Extensive demethylation of the entire genome in endosperm cells, coupled with hypermethylation of non-CG residues (especially CHH sites on transposable elements in the genome of embryonic cells), was reported to drive genetic imprinting in the *Arabidopsis* endosperm and embryo [29], [30]. A similar system appears to operate in pollen [31]. In animals, DNA methylation and demethylation are hormonally regulated to control transcription of the *CYP27B1* gene, which encodes a cytochrome P450 enzyme [32]. The periodic strand-specific methylation and demethylation associated with transcriptional cycling of the *pS2/TFF1* promoter upon estrogen activation [33] suggests that gene expression can be linked directly to DNA methylation status.

In plants, genes, transposons and repetitive sequences were found to be methylated in different densities at various developmental stages, which suggested that the transcription of certain genes is controlled epigenetically [34]–[36]. Indeed, promoter DNA hypermethylation was related to target gene repression in undifferentiated *Arabidopsis* cells [37]. Recently, multiple exposures to drought were found to alter transcriptional responses of a large set of *Arabidopsis* genes mediated probably by H3K4me3 [38].

Here, we not only report an annual pattern of CHH DNA methylation in the promoter regions of the growth-regulating genes *SUR4*, *KCS13* and *ERF6*, but also an inverse correlation between the potential for cotton fiber growth and the degree of cytosine methylation over the course of the year. The expression and methylation data from ovules of growing plants rather than from cultured ovules is reported here because we think that the annual growth potential change is occurring naturally throughout the one-year cycle. Methylation pattern and target gene expression profiles changed more intensely in cultured cotton ovules or fibers. Ovule culture, especially dissection of ovules, altered the *ROS1* gene expression level significantly (data not shown). No CHH site on *ERF6*, *SUR4* and *KCS13* upstream regulatory regions showed significant (at the p<0.05 level) methylation patterning when cotton samples of different development stages were harvested and used for bisulfite sequencing (Figure S4), indicating that the reported growth potential change may function independently of developmental regulations. Our results indicate that CHH DNA methylation/demethylation may constitute a potential novel epigenetic mechanism that regulates growth performance in higher plants over the one-year period. We suggest that a complex epigenetic regulatory network including histone acetylation, histone methylation and CHH DNA methylation, may operate in plants to specifically memorize time-of-day, seasons and time-of-year. This long-term memory system, in conjunction with the vernalization mechanism, may provide a useful tool for the plant to conteract in unfavourable growth conditions. Whole genome bisulfite sequencing and in-depth single molecular DNA methylation analyses are required to further clarify this annual DNA methylation patterning and to point out its biological significance.

### Accession Numbers

A cotton ovule cDNA microarray containing 28,178 UniESTs was deposited to NCBI with GEO accession no. GPL8569. *GhDRM1/2*, *GhCMT3*, *GhDCL3*, *GhDME*, and *GhROS1* were deposited to NCBI with accession nos. from HQ229653 to HQ229657, respectively.

## Supporting Information

Figure S1 **Graphical output of GOEAST analysis to identify enriched gene ontology (GO) end-terms (biological process categories) for genes that are differentially expressed over one year.** Of 235 genes that were preferentially expressed either during summer or during winter (Table S2), 176 were recognized in GO and used for analysis. Each box is labeled by a specific GO identifier and a brief term definition. Significantly enriched GO terms (false discovery rate-corrected P values <0.01; Benjamini and Hochberg, 1995) are in yellow and non-significant terms are in white. The degree of color saturation in each node correlates with the enrichment of the corresponding GO term. Branches of the GO hierarchical tree without at least one significantly enriched GO term were not included. Red arrows represent relationships between two enriched GO terms, black solid arrows indicate one enriched and one unenriched term, and black dashed arrows indicate two unenriched GO terms.
(PDF)

Figure S2 **Production and specificity analysis of antibodies for cotton DRM1/2 and ROS1.** (A) Analyses used to design oligopeptides for producing anti-DRM1/2 (top) and anti-ROS1 (bottom). Rabbits were immunized with oligopeptides indicated by green frames and shown in red letters. Full-length DRM1/2 and the C-terminal 600 amino acids of ROS1 were used for the analysis. (B) Examination of antibody titers by ELISA. Blank, no serum added. Negative, pre-bleed serum added. (C) SDS-PAGE of cotton DRM1/2 (amino acids 1–299; left) and ROS1 (amino acids 106–441; right) expressed in *E. coli*. Both fragments were cloned in pET28a, with 34-amino acid His tags. Total cellular proteins extracted from cotton (cot.) were loaded in both gels. Protein yields are shown before () and after (+) IPTG induction. Arrowheads indicate increased protein production after IPTG induction. (D) Western blotting to confirm specificity of anti-DRM1/2 (left) and anti-ROS1 (right) produced from the oligopeptides depicted as gray shaded areas in (A,B). The predicted DRM1/2 protein contains 636 amino acids with a theoretical MW of 71 kDa, and ROS1 contains 1,758 amino acids with a theoretical MW of 197 kDa (Table S4).
(PDF)

Figure S3 **Bisulfite sequencing of control sequences.** (A) Bisulfite sequencing confirms that the cotton *GheIF2A* coding sequence consistently contains very few methylated sites throughout the year. Genomic DNA samples prepared from May 2010 to February 2011 were used as the template for PCR after bisulfite treatment. The sequence from 509 to 958 bp of JQ922565 was amplified for sequencing reactions. (B) Bisulfite sequencing confirms that the cotton *GhTUB3* upstream region is highly methylated throughout the year. Genomic DNA samples prepared from May 2010 to February 2011 were used as the template for PCR after bisulfite treatment. The sequence from 797 to 1153 bp of JQ922564 was amplified for sequencing reactions.
(PDF)

Figure S4 **Samples harvested from different developmental stages and different tissues showed identical CHH DNA methylation pattern.** (A) Bisulfite sequencing of ERF6 promoter region using DNA samples prepared from ovules grown on cotton plants for 3 DPA and fibers for 10 DPA. (B) Bisulfite sequencing of SUR4 promoter region using DNA samples prepared from ovules grown on cotton plants for 3 DPA and fibers for 10 DPA. (C) Bisulfite sequencing of KCS13 promoter region using DNA samples prepared from ovules grown on cotton plants for 3 DPA and fibers for 10 DPA. All samples were harvested in May 2012.
(PDF)

Table S1 **Microarray accession numbers and correlation coefficients for all hybridizations performed using RNA samples obtained throughout a one-year cycle.**
(PDF)

Table S2 **Genes that were preferentially upregulated from February to August (Class a, 202 genes) and from August to February (Class b, 33 genes).**
(PDF)

Table S3 **Gene ontology (GO) end-terms of significantly upregulated genes.**
(PDF)

Table S4 **Detailed sequence information for genes represented in GO ID: 0006306.**
(PDF)

Table S5 **Relative expression of 20** ***Gossypium hirsutum*** **housekeeping genes based on microarray and QRT-PCR analysis.**
(PDF)

Table S6 **Primers used in the current work.**
(PDF)

Table S7 **Statistical analysis of methylation pattern changes in all the cytosine sites present in the upstream regions shown in** **Figure 3** **.**
(PDF)

Table S8 **Digital intensities of methylation-sensitive endonuclease-digested and full-length fragments.**
(PDF)
