# Supplementary figures and images for: A Potential Role for CHH DNA Methylation in Cotton Fiber Growth Patterns
Source: PLoS One. 2013 Apr 12;8(4):e60547. doi: 10.1371/journal.pone.0060547 (PMC3625195; doi:10.1371/journal.pone.0060547)

Figure S1

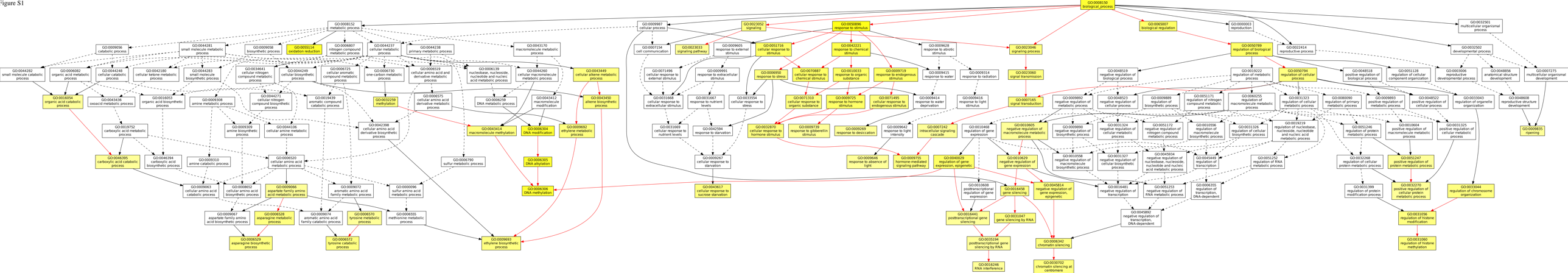

Supplement: Figure S1 — Graphical output of GOEAST analysis to identify enriched gene ontology (GO) end-terms (biological process categories) for genes that are differentially expressed over one year. Of 235 genes that were preferentially expressed either during summer or during winter (Table S2), 176 were recognized in GO and used for analysis. Each box is labeled by a specific GO identifier and a brief term definition. Significantly enriched GO terms (false discovery rate-corrected P values <0.01; Benjamini and Hochberg, 1995) are in yellow and non-significant terms are in white. The degree of color saturation in each node correlates with the enrichment of the corresponding GO term. Branches of the GO hierarchical tree without at least one significantly enriched GO term were not included. Red arrows represent relationships between two enriched GO terms, black solid arrows indicate one enriched and one unenriched term, and black dashed arrows indicate two unenriched GO terms. (PDF) [file pone.0060547.s001.pdf]

# Figure S2

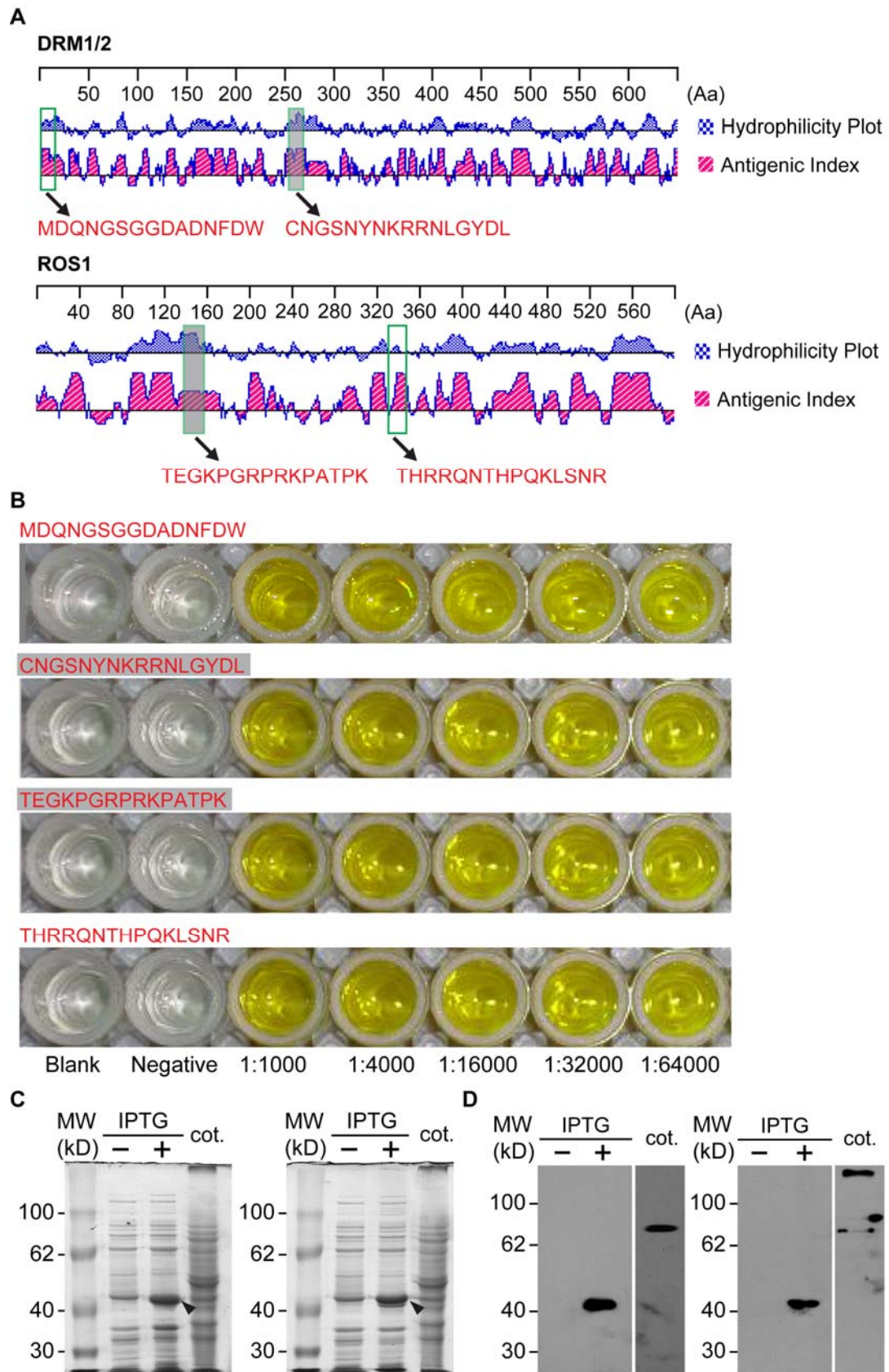

Supplement: Figure S2 — Production and specificity analysis of antibodies for cotton DRM1/2 and ROS1. (A) Analyses used to design oligopeptides for producing anti-DRM1/2 (top) and anti-ROS1 (bottom). Rabbits were immunized with oligopeptides indicated by green frames and shown in red letters. Full-length DRM1/2 and the C-terminal 600 amino acids of ROS1 were used for the analysis. (B) Examination of antibody titers by ELISA. Blank, no serum added. Negative, pre-bleed serum added. (C) SDS-PAGE of cotton DRM1/2 (amino acids 1–299; left) and ROS1 (amino acids 106–441; right) expressed in E. coli. Both fragments were cloned in pET28a, with 34-amino acid His tags. Total cellular proteins extracted from cotton (cot.) were loaded in both gels. Protein yields are shown before () and after (+) IPTG induction. Arrowheads indicate increased protein production after IPTG induction. (D) Western blotting to confirm specificity of anti-DRM1/2 (left) and anti-ROS1 (right) produced from the oligopeptides depicted as gray shaded areas in (A,B). The predicted DRM1/2 protein contains 636 amino acids with a theoretical MW of 71 kDa, and ROS1 contains 1,758 amino acids with a theoretical MW of 197 kDa (Table S4). (PDF) [file pone.0060547.s002.pdf]

Figure S3

**A**

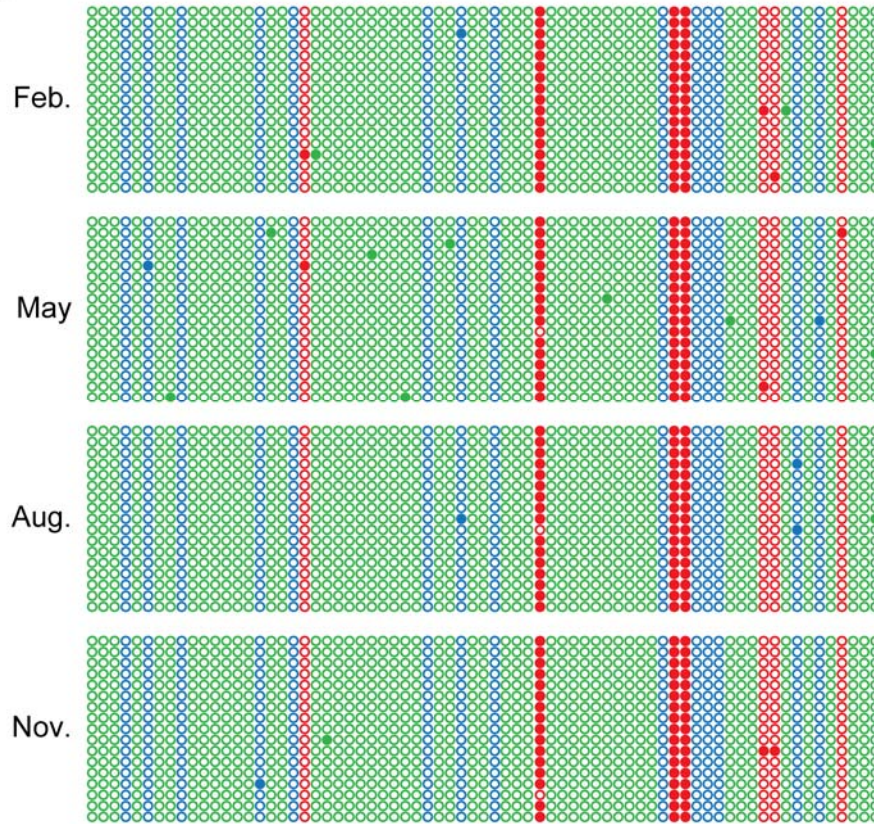

**B**

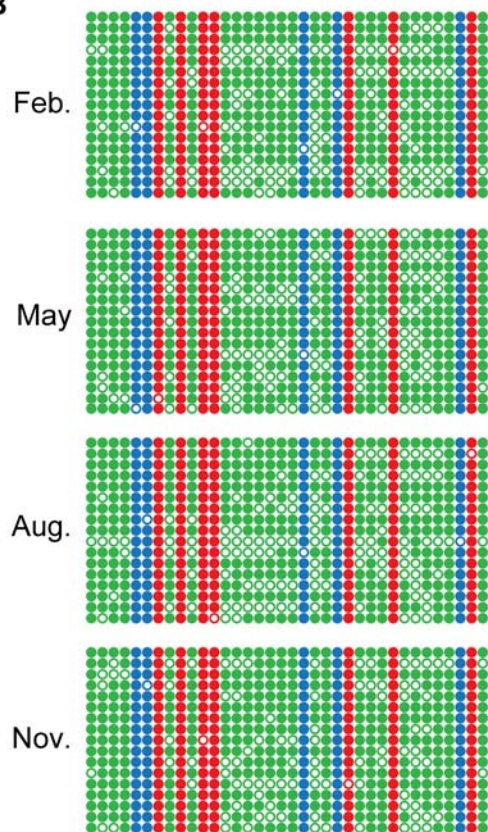

Supplement: Figure S3 — Bisulfite sequencing of control sequences. (A) Bisulfite sequencing confirms that the cotton GheIF2A coding sequence consistently contains very few methylated sites throughout the year. Genomic DNA samples prepared from May 2010 to February 2011 were used as the template for PCR after bisulfite treatment. The sequence from 509 to 958 bp of JQ922565 was amplified for sequencing reactions. (B) Bisulfite sequencing confirms that the cotton GhTUB3 upstream region is highly methylated throughout the year. Genomic DNA samples prepared from May 2010 to February 2011 were used as the template for PCR after bisulfite treatment. The sequence from 797 to 1153 bp of JQ922564 was amplified for sequencing reactions. (PDF) [file pone.0060547.s003.pdf]

**Figure S4**

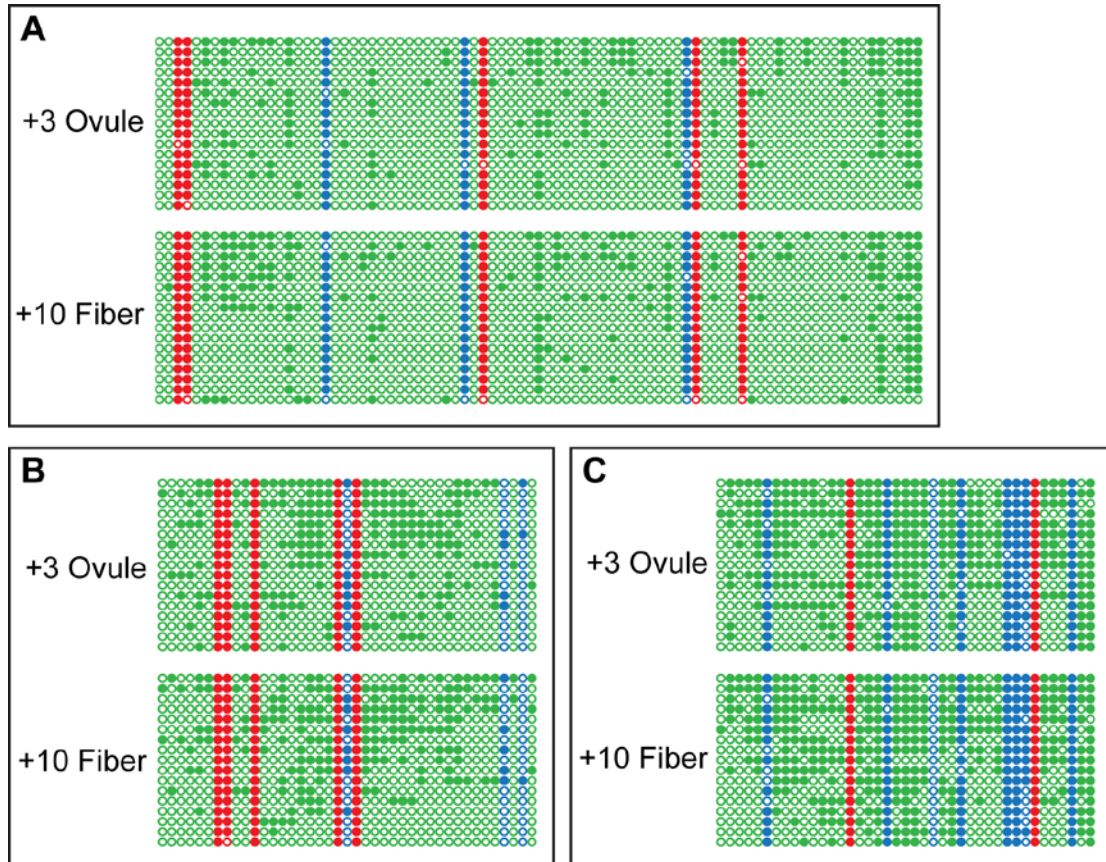

Supplement: Figure S4 — Samples harvested from different developmental stages and different tissues showed identical CHH DNA methylation pattern. (A) Bisulfite sequencing of ERF6 promoter region using DNA samples prepared from ovules grown on cotton plants for 3 DPA and fibers for 10 DPA. (B) Bisulfite sequencing of SUR4 promoter region using DNA samples prepared from ovules grown on cotton plants for 3 DPA and fibers for 10 DPA. (C) Bisulfite sequencing of KCS13 promoter region using DNA samples prepared from ovules grown on cotton plants for 3 DPA and fibers for 10 DPA. All samples were harvested in May 2012. (PDF) [file pone.0060547.s004.pdf]
