# Supplementary material for: A Potential Role for CHH DNA Methylation in Cotton Fiber Growth Patterns
Source: PLoS One. 2013 Apr 12;8(4):e60547. doi: 10.1371/journal.pone.0060547 (PMC3625195; doi:10.1371/journal.pone.0060547)
Supplement: Table S1 — Microarray accession numbers and correlation coefficients for all hybridizations performed using RNA samples obtained throughout a one-year cycle. (PDF) [file pone.0060547.s005.pdf]

Table S1. Microarray Accession Numbers and Correlation Coefficients for All Hybridizations Performed Using RNA Samples Obtained throughout a One-Year Cycle[a]

Sample accession number[b]	Title[c]	Biological coefficient[d]			Technical coefficient
		Set 2	Set 3	Set 4	
GSM419228	cDNA array May / CR set 1	0.9677	0.9732	0.9699	0.9650
GSM419333	cDNA array May / CR set 1 (dye swap)				
GSM419334	cDNA array May / CR set 2		0.9709	0.9825	0.9739
GSM419335	cDNA array May / CR set 2 (dye swap)				
GSM419336	cDNA array May / CR set 3			0.9669	0.9350
GSM419337	cDNA array May / CR set 3 (dye swap)				
GSM419338	cDNA array May / CR set 4				0.9723
GSM419340	cDNA array May / CR set 4 (dye swap)				
GSM419341	cDNA array August / CR set 1	0.9882	0.9861	0.8681	0.9570
GSM419343	cDNA array August / CR set 1 (dye swap)				
GSM419345	cDNA array August / CR set 2		0.9891	0.8620	0.9815
GSM419346	cDNA array August / CR set 2 (dye swap)				
GSM419348	cDNA array August / CR set 3			0.8772	0.9821
GSM419349	cDNA array August / CR set 3 (dye swap)				
GSM419351	cDNA array August / CR set 4				0.9776
GSM419352	cDNA array August / CR set 4 (dye swap)				
GSM419428	cDNA array November / CR set 1	0.9798	0.9648	0.9593	0.9620

GSM419429	cDNA array November / CR set 1 (dye swap)				
GSM419430	cDNA array November / CR set 2		0.9793	0.9770	0.9710
GSM419431	cDNA array November / CR set 2 (dye swap)				
GSM419432	cDNA array November / CR set 3			0.9772	0.9692
GSM419433	cDNA array November / CR set 3 (dye swap)				
GSM419434	cDNA array November / CR set 4				0.9723
GSM419435	cDNA array November / CR set 4 (dye swap)				
GSM419436	cDNA array February / CR set 1	0.9385	0.9419	0.9128	0.9529
GSM419437	cDNA array February / CR set 1 (dye swap)				
GSM419438	cDNA array February / CR set 2		0.9557	0.9238	0.9572
GSM419439	cDNA array February / CR set 2 (dye swap)				
GSM419440	cDNA array February / CR set 3			0.8980	0.9719
GSM419441	cDNA array February / CR set 3 (dye swap)				
GSM419442	cDNA array February / CR set 4				0.9773
GSM419443	cDNA array February / CR set 4 (dye swap)				

[a]All microarray data were submitted to the National Center for Biotechnology Information GEO database in MIAME format (platform title “28,178 cotton cDNA array”; accession no. GPL8569).

[b]All 32 samples were deposited to GEO (series title “cDNA expression profiles of 7-dpa wild-type upland cotton (*Gossypium hirsutum* cv. Xuzhou142) ovules over the 1-year cycle”, serial accession no. GSE16802).

[c]May, August, November, and February samples derived from 7-dpa wild-type upland cotton ovules; CR, common reference, an equal mixture of May, August, November, and February RNA samples; sets 1–4, four biological replicates.

[d]We used four independent RNA samples as biological replicates and used swap-dye experiments of each sample as the technical replicates. The coefficients of both biological and technical replications were obtained by applying statistical tools in the R environment, using a Bioconductor package. We performed LOWESS normalization on the technical replicates and only those microarray spots that passed the test were used for subsequent statistical analysis to obtain the biological coefficients.
