# Supplementary material for: A Potential Role for CHH DNA Methylation in Cotton Fiber Growth Patterns
Source: PLoS One. 2013 Apr 12;8(4):e60547. doi: 10.1371/journal.pone.0060547 (PMC3625195; doi:10.1371/journal.pone.0060547)
Supplement: Table S2 — Genes that were preferentially upregulated from February to August (Class a, 202 genes) and from August to February (Class b, 33 genes). (PDF) [file pone.0060547.s006.pdf]

Table S2. Genes that Were Preferentially Upregulated[a] from February to August (Class a, 202 Genes) and from August to February (Class b, 33 Genes)

Class a	Array ID.	May / CR	Aug / CR	Nov / CR	Feb / CR	Aug / Feb	P	Annotation
	*28k_244_C06	0.90 ± 0.09	3.26 ± 0.25	1.21 ± 0.09	0.67 ± 0.29	4.91 ± 1.40	6.0E-05	PR protein class 10
	*28k_222_G10	0.92 ± 0.06	2.71 ± 0.30	1.21 ± 0.17	0.64 ± 0.06	4.27 ± 0.39	1.99E-07	ethylene-responsive element binding protein 6
	*28k_180_F07	1.00 ± 0.05	2.65 ± 0.16	1.10 ± 0.07	0.60 ± 0.05	4.39 ± 0.29	3.42E-08	ADH1 (ALCOHOL DEHYDROGENASE 1)
	28k_107_E01	0.97 ± 0.05	2.53 ± 0.15	1.17 ± 0.06	0.61 ± 0.07	4.18 ± 0.29	1.44E-08	Alcohol dehydrogenase
	*28k_155_B03	1.02 ± 0.07	2.58 ± 0.23	1.17 ± 0.08	0.68 ± 0.08	3.80 ± 0.38	4.61E-06	alcohol dehydrogenase-like protein
	*28k_142_D06	1.02 ± 0.07	2.46 ± 0.44	1.19 ± 0.29	0.66 ± 0.14	3.73 ± 0.67	1.5E-04	oxidoreductase, zinc-binding dehydrogenase family
	*28k_214_E09	0.92 ± 0.10	4.61 ± 0.52	1.00 ± 0.06	0.61 ± 0.19	7.59 ± 1.00	3.0E-05	glycoside hydrolase family 28 protein
	*28k_029_B12	1.01 ± 0.07	2.20 ± 0.20	1.00 ± 0.08	0.69 ± 0.05	3.18 ± 0.30	1.0E-05	plastid-lipid associated protein PAP
	*28k_218_A12	1.00 ± 0.25	1.66 ± 0.13	1.00 ± 0.10	0.76 ± 0.36	2.19 ± 0.54	2.9.0E-04	nucleotidyltransferase family protein
	28k_188_E04	1.29 ± 0.24	4.12 ± 0.39	1.56 ± 0.21	0.65 ± 0.18	6.30 ± 0.65	1.0E-05	No hits
	*28k_294_A01	1.20 ± 0.04	3.64 ± 0.29	1.45 ± 0.24	0.68 ± 0.14	5.38 ± 0.58	1.24E-08	AAP3 ; amino acid transmembrane transporter
	*28k_234_C05	1.31 ± 0.05	3.25 ± 0.13	1.32 ± 0.11	0.74 ± 0.22	4.38 ± 0.68	2.6E-04	ATTPS11 (Arabidopsis thaliana trehalose phosphatase/synthase 11)
	28k_298_H01	1.06 ± 0.05	1.74 ± 0.14	1.13 ± 0.08	0.84 ± 0.18	2.07 ± 0.34	1.69E-06	similar to unknown protein (TAIR:AT3G43850.1)
	*28k_290_C10	1.19 ± 0.08	3.27 ± 0.24	1.27 ± 0.15	0.56 ± 0.22	5.79 ± 0.75	3.0E-05	ANAC100/ATNAC5 (Arabidopsis NAC domain containing protein 100)
	28k_168_G07	1.12 ± 0.06	2.30 ± 0.30	1.14 ± 0.13	0.67 ± 0.14	3.43 ± 0.57	4.0E-05	binding/catalytic/transcription repressor
	*28k_250_H01	1.10 ± 0.10	1.76 ± 0.20	1.10 ± 0.10	0.80 ± 0.09	2.20 ± 0.32	5.0E-05	ethylene-responsive family protein
	*28k_289_D11	1.08 ± 0.04	5.01 ± 0.63	1.09 ± 0.09	0.52 ± 0.14	9.56 ± 1.07	1.9E-04	asparagine synthetase
	*28k_187_H04	1.09 ± 0.06	1.69 ± 0.12	1.02 ± 0.09	0.81 ± 0.13	2.08 ± 0.30	1.31E-06	ALDH6B2 (Aldehyde dehydrogenase 6B2)
	*28k_297_F01	1.33 ± 0.16	2.52 ± 0.29	1.09 ± 0.10	0.66 ± 0.12	3.84 ± 0.65	5.0E-05	HAT22 (homeobox-leucine zipper protein 22)

*28k_222_E11	1.25 ± 0.07	2.18 ± 0.14	1.11 ± 0.09	0.69 ± 0.09	3.16 ± 0.38	2.0E-05	DME (DEMETER)
*28k_269_C06	1.17 ± 0.09	1.70 ± 0.19	1.09 ± 0.09	0.80 ± 0.22	2.12 ± 0.39	8.0E-05	GNS1/SUR4 membrane protein family
28k_198_A11	0.97 ± 0.13	4.07 ± 0.21	1.09 ± 0.14	0.84 ± 0.20	4.85 ± 0.50	1.53E-06	No hits
*28k_233_D02	0.97 ± 0.13	2.29 ± 0.25	1.09 ± 0.13	0.89 ± 0.19	2.56 ± 0.42	2.1E-04	short-chain dehydrogenase/reductase (SDR)
*28k_084_F03	1.03 ± 0.18	2.24 ± 0.38	1.11 ± 0.09	0.86 ± 0.12	2.60 ± 0.53	4.5E-04	PIL5 (PHYTOCHROME INTERACTING FACTOR 3-LIKE 5)
*28k_232_C08	1.13 ± 0.09	2.84 ± 0.16	0.93 ± 0.05	0.92 ± 0.16	3.10 ± 0.29	3.27E-07	AERO1 (ARABIDOPSIS ENDOPLASMIC RETICULUM OXIDOREDUCTINS 1)
*28k_273_G02	1.08 ± 0.15	2.13 ± 0.23	1.00 ± 0.09	0.93 ± 0.23	2.29 ± 0.50	5.1E-04	ACX4 (ACYL-COA OXIDASE 4); oxidoreductase
*28k_198_C08	0.85 ± 0.11	4.54 ± 0.83	0.80 ± 0.05	0.72 ± 0.10	6.29 ± 1.04	1.0E-05	universal stress protein (USP) family protein[Arabidopsis thaliana]
28k_203_E04	0.88 ± 0.07	2.87 ± 0.53	0.90 ± 0.09	0.86 ± 0.10	3.35 ± 0.67	5.0E-05	similar to unknown protein (TAIR:AT5G42690.2)
*28k_209_F03	1.00 ± 0.04	2.17 ± 0.27	0.91 ± 0.11	0.78 ± 0.09	2.78 ± 0.37	2.76E-06	CK1 (casein kinase 1-like protein 1)
*28k_133_G05	1.00 ± 0.10	1.88 ± 0.24	0.89 ± 0.08	0.88 ± 0.12	2.15 ± 0.41	1.6E-04	ARABIDOPSIS THALIANA EXPANSIN A1
28k_196_D08	1.10 ± 0.06	1.78 ± 0.15	0.89 ± 0.05	0.88 ± 0.10	2.01 ± 0.26	1.0E-05	predicted protein
*28k_134_A07	1.24 ± 0.07	2.05 ± 0.18	0.95 ± 0.13	0.61 ± 0.38	3.35 ± 1.52	4.8E-04	GDSL-motif lipase/hydrolase family protein
*28k_233_D07	1.26 ± 0.08	1.98 ± 0.21	0.96 ± 0.17	0.71 ± 0.36	2.81 ± 1.35	4.2E-04	3-ketoacyl-CoA synthase
*28k_083_C02	1.08 ± 0.11	1.61 ± 0.12	0.90 ± 0.09	0.75 ± 0.13	2.15 ± 0.34	1.1E-04	ATPK19; ARABIDOPSIS THALIANA PROTEIN KINASE
*28k_135_E06	1.13 ± 0.07	1.52 ± 0.09	0.88 ± 0.08	0.76 ± 0.19	2.01 ± 0.41	2.0E-05	Serine/threonine-protein kinase AtPK19
28k_224_H07	0.99 ± 0.07	2.14 ± 0.19	0.89 ± 0.08	0.62 ± 0.14	3.45 ± 0.50	1.31E-06	MEE14 (maternal effect embryo arrest 14)
*28k_107_D10	0.94 ± 0.13	1.83 ± 0.26	0.95 ± 0.12	0.72 ± 0.13	2.56 ± 0.47	8.0E-04	pathogenesis-related protein 10
28k_215_E01	0.95 ± 0.11	1.54 ± 0.12	0.97 ± 0.08	0.74 ± 0.13	2.08 ± 0.29	2.0E-05	similar to unknown protein (TAIR:AT4G27435.1)

*28k_077_B05	0.92 ± 0.06	1.61 ± 0.13	0.90 ± 0.04	0.70 ± 0.03	2.29 ± 0.18	2.91E-07	ATCTH (Arabidopsis thaliana Cys3His zinc finger protein)
28k_012_A12	0.98 ± 0.12	1.46 ± 0.14	0.94 ± 0.07	0.73 ± 0.13	2.02 ± 0.39	3.0E-05	MT2A (METALLOTHIONEIN 2A)
*28k_173_D03	0.86 ± 0.03	2.36 ± 0.12	1.07 ± 0.08	0.53 ± 0.13	4.44 ± 0.51	2.18E-07	haloacid dehalogenase-like hydrolase family
28k_220_C08	0.86 ± 0.03	2.32 ± 0.17	1.15 ± 0.10	0.53 ± 0.14	4.40 ± 0.56	4.61E-07	EFE (ETHYLENE FORMING ENZYME)
*28k_279_C02	0.90 ± 0.04	2.97 ± 0.30	1.19 ± 0.11	0.49 ± 0.21	6.08 ± 1.06	2.6E-04	PR protein class 10
*28k_287_H11	0.91 ± 0.04	1.73 ± 0.13	1.09 ± 0.06	0.72 ± 0.20	2.38 ± 0.54	2.0 E-05	phospholipase C
28k_274_D03	0.82 ± 0.05	2.16 ± 0.20	1.08 ± 0.06	0.63 ± 0.16	3.40 ± 0.50	4.42E-07	transcription factor
*28k_283_F05	0.92 ± 0.07	2.23 ± 0.28	1.01 ± 0.12	0.61 ± 0.06	3.67 ± 0.44	4.26E-06	HGO (Homogentisate 1,2-Dioxygenase)
*28k_198_C01	0.86 ± 0.07	5.98 ± 0.27	1.12 ± 0.03	0.35 ± 0.11	16.95 ± 1.10	1.67E-09	GAST1 PROTEIN HOMOLOG 4
*28k_167_H09	0.92 ± 0.03	2.42 ± 0.20	1.20 ± 0.06	0.50 ± 0.08	4.79 ± 0.53	1.28E-08	AMP binding / acetate-CoA ligase
28k_054_B07	0.84 ± 0.04	2.56 ± 0.19	1.18 ± 0.10	0.43 ± 0.14	5.93 ± 0.91	2.35E-08	catalytic/ hydrolase
*28k_178_A10	0.85 ± 0.08	2.51 ± 0.15	1.05 ± 0.08	0.42 ± 0.13	5.92 ± 0.92	4.70E-08	haloacid dehalogenase-like hydrolase family
*28k_152_F03	0.80 ± 0.05	2.35 ± 0.10	1.06 ± 0.07	0.45 ± 0.14	5.20 ± 0.86	3.68E-08	haloacid dehalogenase-like hydrolase family
*28k_101_A07	0.97 ± 0.14	1.48 ± 0.14	1.02 ± 0.11	0.68 ± 0.16	2.17 ± 0.47	6.1E-04	4-hydroxyphenylpyruvate dioxygenase
28k_158_G01	0.99 ± 0.06	2.07 ± 0.30	0.98 ± 0.03	0.64 ± 0.10	3.24 ± 0.49	3.0E-05	MEE14 (maternal effect embryo arrest 14)
*28k_289_E06	1.03 ± 0.05	1.99 ± 0.13	0.99 ± 0.04	0.63 ± 0.16	3.13 ± 0.48	5.69E-07	MEE14 (maternal effect embryo arrest 14)
28k_214_C07	1.07 ± 0.04	3.05 ± 0.23	1.04 ± 0.08	0.49 ± 0.14	6.26 ± 1.01	3.39E-08	zinc-binding family
*28k_208_B02	1.08 ± 0.11	2.45 ± 0.29	1.08 ± 0.22	0.53 ± 0.24	4.62 ± 1.12	4.0E-05	haloacid dehalogenase-like hydrolase family protein
*28k_163_D09	1.20 ± 0.12	4.72 ± 0.86	1.54 ± 0.21	0.83 ± 0.41	5.69 ± 1.65	1.2E-04	SAG21 (Senescence-Associated Gene 21)
*28k_106_D08	1.12 ± 0.06	2.71 ± 0.26	1.15 ± 0.07	0.92 ± 0.25	2.93 ± 0.47	1.99E-06	Arabidopsis NAC domain containing protein 83
28k_297_D02	0.98 ± 0.04	2.49 ± 0.17	1.33 ± 0.08	0.96 ± 0.24	2.59 ± 0.40	2.0E-05	unnamed protein product

*28k_250_B11	1.00 ± 0.06	1.67 ± 0.17	1.41 ± 0.12	0.81 ± 0.09	2.05 ± 0.28	1.0E-05	zinc finger (B-box type) family
*28k_097_E06	1.03 ± 0.09	1.73 ± 0.19	1.39 ± 0.15	0.87 ± 0.08	2.00 ± 0.29	9.0E-05	ABI2 (ABA INSENSITIVE 2)
*28k_303_B02	1.57 ± 0.09	2.17 ± 0.21	1.61 ± 0.11	0.64 ± 0.19	3.40 ± 1.02	2.1E-04	ROS1 (repressor of silencing 1)
*28k_169_D11	1.39 ± 0.09	2.18 ± 0.27	1.43 ± 0.30	0.73 ± 0.10	2.98 ± 0.44	6.0E-05	EBF1 (Ein3-Binding F Box Protein 1)
*28k_171_A08	1.23 ± 0.06	1.66 ± 0.06	1.19 ± 0.12	0.82 ± 0.08	2.02 ± 0.17	1.63E-07	EIN3 (ETHYLENE-INSENSITIVE3)
*28k_225_E02	1.60 ± 0.62	2.02 ± 0.10	1.40 ± 0.06	0.83 ± 0.12	2.43 ± 0.29	7.92E-08	SKU5; copper ion binding
*28k_219_E04	1.20 ± 0.34	2.42 ± 0.35	1.17 ± 0.07	0.46 ± 0.12	5.25 ± 0.76	5.76E-07	AAE7/ACN1 (Acyl-Activating Enzyme 7)
*28k_239_A06	1.20 ± 0.09	2.01 ± 0.50	1.09 ± 0.10	0.55 ± 0.20	3.64 ± 1.00	3.5E-04	asparagine synthetase
*28k_293_C12	1.20 ± 0.18	2.37 ± 0.28	1.07 ± 0.13	0.40 ± 0.13	5.88 ± 1.17	5.7E-04	hypothetical protein
*28k_212_G04	1.10 ± 0.10	1.71 ± 0.21	0.98 ± 0.19	0.59 ± 0.10	2.92 ± 0.49	4.0E-05	LOX1 (Lipoxygenase 2)
*28k_263_H06	1.31 ± 0.06	2.89 ± 0.22	1.46 ± 0.20	0.48 ± 0.09	6.05 ± 0.54	5.77E-08	Putative reticuline oxidase-like protein
*28k_108_E08	1.23 ± 0.11	2.83 ± 0.30	1.52 ± 0.12	0.42 ± 0.07	6.68 ± 0.57	1.38E-06	second sucrose synthase
28k_287_C07	1.14 ± 0.20	2.18 ± 0.18	1.32 ± 0.16	0.54 ± 0.06	4.00 ± 0.51	3.0E-05	ATGDU2 (ARABIDOPSIS THALIANA GLUTAMINE DUMPER 2)
28k_119_H09	1.06 ± 0.05	1.78 ± 0.19	1.26 ± 0.13	0.61 ± 0.18	2.91 ± 0.71	1.0E-05	LBD41 (LOB DOMAIN-CONTAINING PROTEIN 41)
28k_235_C06	1.08 ± 0.05	3.88 ± 0.38	1.31 ± 0.17	0.39 ± 0.21	9.89 ± 4.09	1.0E-05	No apical meristem (NAM) protein family
*28k_171_B10	1.08 ± 0.06	2.33 ± 0.25	1.29 ± 0.09	0.56 ± 0.12	4.19 ± 0.56	8.83E-07	beta-fructosidase (BFRUCT3) / beta-fructofuranosidase
*28k_267_B03	1.13 ± 0.08	1.86 ± 0.20	1.12 ± 0.06	0.63 ± 0.20	2.94 ± 0.78	1.0E-05	ADH1 (ALCOHOL DEHYDROGENASE 1)
28k_227_F02	1.08 ± 0.06	1.53 ± 0.24	1.07 ± 0.07	0.75 ± 0.13	2.06 ± 0.40	9.0E-05	leucine-rich repeat family
*28k_117_G12	1.08 ± 0.08	1.60 ± 0.13	1.12 ± 0.07	0.72 ± 0.11	2.23 ± 0.33	2.0E-05	BAS1/CYP734A1 (PHYB ACTIVATION TAGGED SUPPRESSOR 1)

*28k_246_D05	1.40 ± 0.14	1.53 ± 0.13	1.17 ± 0.06	0.63 ± 0.16	2.44 ± 0.59	5.2E-04	transformer serine/arginine-rich ribonucleoprotein
*28k_224_D07	1.34 ± 0.12	1.63 ± 0.12	1.41 ± 0.20	0.52 ± 0.37	3.15 ± 1.93	3.0E-05	putative xyloglucan endotransglycosylase
*28k_193_A05	1.18 ± 0.10	1.43 ± 0.09	1.18 ± 0.10	0.66 ± 0.06	2.15 ± 0.24	1.79E-06	EIN3 (ETHYLENE-INSENSITIVE3)
*28k_169_C02	1.14 ± 0.13	1.32 ± 0.15	1.15 ± 0.08	0.66 ± 0.04	2.01 ± 0.23	1.0E-05	prenylated rab acceptor (PRA1)
*28k_176_A01	1.39 ± 0.10	2.48 ± 0.18	1.09 ± 0.09	0.24 ± 0.13	10.47 ± 2.27	1.07E-07	SAG21 (SENESCENCE-ASSOCIATED GENE 21)
*28k_257_B05	1.21 ± 0.11	1.42 ± 0.06	1.03 ± 0.13	0.55 ± 0.09	2.56 ± 0.40	3.36E-07	similar to loricrin-related (TAIR:AT5G64550.1)
28k_284_E10	1.22 ± 0.09	1.37 ± 0.09	0.98 ± 0.17	0.67 ± 0.12	2.04 ± 0.27	5.2E-04	senescence-associated family protein
*28k_222_B03	1.10 ± 0.07	1.52 ± 0.12	0.98 ± 0.10	0.54 ± 0.10	2.82 ± 0.59	1.18E-06	ACX3 (ACYL-COA OXIDASE 3); acyl-CoA oxidase
*28k_256_B01	1.09 ± 0.05	1.54 ± 0.12	0.93 ± 0.04	0.49 ± 0.07	3.18 ± 0.46	2.32E-07	LOX1 (Lipoxygenase 1); lipoxygenase
*28k_172_B03	1.06 ± 0.06	1.37 ± 0.06	0.97 ± 0.05	0.61 ± 0.12	2.24 ± 0.41	4.26E-07	LOX1 (Lipoxygenase 3); lipoxygenase
28k_279_D06	1.09 ± 0.06	1.47 ± 0.12	0.97 ± 0.07	0.62 ± 0.09	2.39 ± 0.34	1.0E-05	zinc-binding family
28k_046_H09	1.02 ± 0.08	1.39 ± 0.09	0.99 ± 0.06	0.65 ± 0.05	2.16 ± 0.21	2.21E-06	similar to CBS domain-containing protein (TAIR:AT2G14520.1)
*28k_072_B11	1.08 ± 0.04	2.43 ± 0.23	1.58 ± 0.13	0.47 ± 0.07	5.21 ± 0.50	2.32E-07	CIPK6 (CBL-INTERACTING PROTEIN KINASE 6)
*28k_074_F06	1.05 ± 0.07	2.10 ± 0.15	1.58 ± 0.18	0.47 ± 0.17	4.47 ± 0.99	1.0E-05	nodulin MtN21 family protein
*28k_203_D01	1.07 ± 0.11	1.68 ± 0.11	1.32 ± 0.09	0.57 ± 0.05	2.94 ± 0.27	4.31E-08	CIPK6 (CBL-INTERACTING PROTEIN KINASE 6)
*28k_090_A08	1.03 ± 0.09	1.45 ± 0.15	1.14 ± 0.11	0.66 ± 0.09	2.20 ± 0.35	4.0E-05	leucine-rich repeat family
*28k_287_E06	1.05 ± 0.07	1.36 ± 0.09	1.13 ± 0.07	0.68 ± 0.28	2.00 ± 0.77	3.3E-04	xyloglucan:xyloglucosyl transferase/endo-xyloglucan transferase

*28k_292_C01	1.15 ± 0.08	1.62 ± 0.15	1.44 ± 0.17	0.42 ± 0.10	3.87 ± 0.86	5.5E-04	ALDH3H1 (Aldehyde Dehydrogenase 4)
*28k_289_D12	1.08 ± 0.09	1.53 ± 0.13	1.34 ± 0.10	0.55 ± 0.09	2.76 ± 0.39	1.18E-07	oxysterol-binding family
*28k_002_A09	1.03 ± 0.09	1.42 ± 0.14	1.24 ± 0.10	0.64 ± 0.12	2.22 ± 0.42	1.0E-05	COB (COBRA)
28k_256_G10	0.94 ± 0.15	1.46 ± 0.07	1.25 ± 0.11	0.60 ± 0.10	2.43 ± 0.45	1.0E-05	similar to unknown protein (TAIR:AT1G21670)
*28k_228_G06	1.01 ± 0.09	1.38 ± 0.06	1.20 ± 0.07	0.59 ± 0.06	2.33 ± 0.23	3.14E-07	CCR4-NOT transcription complex protein
28k_077_E10	0.98 ± 0.06	1.36 ± 0.08	1.31 ± 0.08	0.58 ± 0.10	2.32 ± 0.32	4.24E-06	auxin/aluminum-responsive protein
*28k_085_C01	0.87 ± 0.06	2.12 ± 0.20	1.42 ± 0.14	0.43 ± 0.15	4.93 ± 1.13	8.44E-07	EFE (ETHYLENE FORMING ENZYME)
*28k_292_F10	0.94 ± 0.11	1.43 ± 0.12	1.16 ± 0.08	0.70 ± 0.10	2.04 ± 0.40	1.7E-04	DNA binding / zinc ion binding
28k_225_H12	0.91 ± 0.09	1.57 ± 0.19	1.25 ± 0.08	0.71 ± 0.16	2.21 ± 0.49	1.5E-04	AILP1 (aluminium-induced protein with YGL and LRDR motifs)
28k_273_H06	0.97 ± 0.14	1.48 ± 0.09	1.54 ± 0.12	0.60 ± 0.21	2.45 ± 0.72	2.0E-05	ATSIP2 (ARABIDOPSIS THALIANA SEED IMBIBITION 2)
*28k_131_C10	0.91 ± 0.07	1.62 ± 0.12	1.57 ± 0.09	0.53 ± 0.15	3.05 ± 1.04	4.41E-06	ATSIP2 (ARABIDOPSIS THALIANA SEED IMBIBITION 2);
28k_279_E01	0.95 ± 0.05	1.46 ± 0.06	1.39 ± 0.07	0.62 ± 0.10	2.35 ± 0.32	5.24E-07	auxin/aluminum-responsive protein
28k_273_F12	0.95 ± 0.03	1.39 ± 0.08	1.31 ± 0.10	0.66 ± 0.08	2.12 ± 0.28	1.52E-06	auxin/aluminum-responsive protein
*28k_151_G03	1.00 ± 0.08	1.39 ± 0.14	1.76 ± 0.23	0.58 ± 0.10	2.38 ± 0.39	1.0E-05	ATSEH (Arabidopsis thaliana soluble epoxide hydrolase)
*28k_294_A08	0.98 ± 0.06	1.38 ± 0.15	1.59 ± 0.19	0.59 ± 0.09	2.36 ± 0.76	7.3E-04	serine/threonine protein kinase
*28k_214_F06	0.96 ± 0.05	1.89 ± 0.13	1.59 ± 0.12	0.60 ± 0.15	3.16 ± 0.88	1.10E-06	ATSIP2 (ARABIDOPSIS THALIANA SEED IMBIBITION 2)
*28k_242_A09	1.09 ± 0.11	1.55 ± 0.12	1.43 ± 0.11	0.70 ± 0.09	2.22 ± 0.27	3.0E-05	CIPK6 (CBL-INTERACTING PROTEIN KINASE 6)

*28k_251_C02	1.18 ± 0.03	1.39 ± 0.08	1.42 ± 0.12	0.68 ± 0.09	2.04 ± 0.27	1.52E-06	protein phosphatase 2C/PP2C
*28k_072_G05	0.75 ± 0.08	1.98 ± 0.25	0.84 ± 0.06	0.50 ± 0.07	3.99 ± 0.43	4.62E-06	putative DnaJ protein
*28k_162_B04	0.89 ± 0.07	1.49 ± 0.09	0.87 ± 0.06	0.59 ± 0.06	2.50 ± 0.24	1.97E-07	zinc finger (CCCH-type) family
*28k_191_C02	0.84 ± 0.03	2.41 ± 0.11	1.20 ± 0.04	0.35 ± 0.19	6.86 ± 2.74	1.68E-07	cysteine proteinase, putative
28k_250_A04	0.89 ± 0.11	1.60 ± 0.09	1.06 ± 0.08	0.49 ± 0.10	3.29 ± 0.50	3.67E-10	dormancy/auxin associated family protein
*28k_268_E03	0.83 ± 0.04	1.49 ± 0.16	1.01 ± 0.08	0.63 ± 0.06	2.38 ± 0.30	1.0E-05	heat shock protein binding / unfolded protein binding
*28k_217_E04	0.87 ± 0.10	1.44 ± 0.19	1.10 ± 0.13	0.51 ± 0.12	2.82 ± 0.87	2.0E-05	protease inhibitor/seed storage/lipid transfer protein (LTP) family
28k_220_B06	0.85 ± 0.05	1.38 ± 0.09	1.14 ± 0.05	0.50 ± 0.19	2.77 ± 0.62	1.0E-05	dormancy/auxin associated family protein
*28k_177_A08	1.06 ± 0.08	1.68 ± 0.12	1.30 ± 0.07	0.30 ± 0.09	5.54 ± 1.40	8.29E-08	PREDICTED: hypothetical protein
28k_143_C04	1.16 ± 0.11	1.56 ± 0.07	1.15 ± 0.08	0.43 ± 0.16	3.63 ± 0.80	9.36E-08	predicted protein
*28k_191_E10	0.95 ± 0.05	2.39 ± 0.19	1.25 ± 0.10	0.30 ± 0.18	7.89 ± 2.27	4.30E-08	ACC1 (acetyl-coenzyme a carboxylase 1)
*28k_191_C03	0.99 ± 0.03	1.59 ± 0.10	1.17 ± 0.05	0.44 ± 0.08	3.62 ± 0.55	1.33E-07	cysteine proteinase
*28k_186_G01	1.00 ± 0.05	1.36 ± 0.09	1.10 ± 0.09	0.62 ± 0.06	2.19 ± 0.22	5.52E-07	ABF2 (ABSCISIC ACID RESPONSIVE ELEMENTS-BINDING FACTOR 2)
*28k_196_B05	1.01 ± 0.03	1.78 ± 0.09	0.93 ± 0.05	0.29 ± 0.04	6.11 ± 0.76	5.79E-08	ATARFA1E (ADP-RIBOSYLATION FACTOR A1E); GTP binding / phospholipase
*28k_151_C03	1.04 ± 0.05	1.39 ± 0.11	0.98 ± 0.07	0.49 ± 0.14	2.82 ± 0.80	2.0E-05	CDA1 (CYTIDINE DEAMINASE 1)
*28k_224_E12	0.89 ± 0.21	2.25 ± 0.14	1.17 ± 0.20	0.16 ± 0.14	13.83 ± 3.38	1.55E-06	peroxidase 30 (PER30) (P30) (PRXR9)
*28k_111_C12	0.99 ± 0.09	1.35 ± 0.21	1.10 ± 0.14	0.50 ± 0.07	2.68 ± 0.44	1.0E-04	chloroplast nucleoid DNA-binding protein
28k_181_H07	0.97 ± 0.06	1.38 ± 0.16	1.04 ± 0.08	0.56 ± 0.17	2.47 ± 0.74	1.0E-05	wound-responsive family protein
28k_142_F11	0.96 ± 0.06	1.34 ± 0.07	1.02 ± 0.08	0.56 ± 0.07	2.40 ± 0.26	1.51E-06	zinc-binding family

*28k_187_F02	0.86 ± 0.08	1.40 ± 0.10	1.02 ± 0.13	0.38 ± 0.12	3.70 ± 1.31	3.0E-05	C/VIF1 (Cell Wall / Vacuolar Inhibitor of Fructosidase 1)
*28k_184_G07	0.98 ± 0.21	1.41 ± 0.06	1.03 ± 0.11	0.38 ± 0.07	3.70 ± 0.54	1.00E-08	LKR (Saccharopine Dehydrogenase)
*28k_195_C06	0.98 ± 0.07	1.19 ± 0.10	0.98 ± 0.08	0.59 ± 0.09	2.01 ± 0.37	1.0E-05	ATNADP-ME2 (NADP-MALIC ENZYME 2)
28k_085_B04	0.97 ± 0.03	1.27 ± 0.11	0.99 ± 0.08	0.55 ± 0.06	2.33 ± 0.44	8.01E-07	predicted protein
28k_132_A05	0.96 ± 0.10	1.24 ± 0.14	0.99 ± 0.08	0.59 ± 0.16	2.11 ± 0.61	3.0E-05	wound-responsive family
*28k_175_B06	0.98 ± 0.02	1.55 ± 0.09	0.91 ± 0.05	0.50 ± 0.07	3.08 ± 0.37	2.58E-07	AUXIN-REPRESSED 12.5 KD PROTEIN
*28k_164_B07	0.91 ± 0.07	1.35 ± 0.10	0.90 ± 0.07	0.55 ± 0.07	2.45 ± 0.28	9.59E-07	zinc finger (CCCH-type) family
*28k_225_G08	1.17 ± 0.05	1.70 ± 0.29	1.35 ± 0.14	0.19 ± 0.02	8.89 ± 0.80	2.0E-05	ATHB21/HB-2 (homeobox-2); DNA binding / transcription factor
*28k_293_E04	1.04 ± 0.11	1.32 ± 0.18	1.10 ± 0.12	0.43 ± 0.17	3.08 ± 1.25	5.0E-05	nodulin MtN3 family protein
*28k_292_G04	1.05 ± 0.08	1.24 ± 0.10	1.07 ± 0.09	0.52 ± 0.07	2.39 ± 0.35	1.05E-06	AUX1 (AUXIN RESISTANT 1); amino acid transmembrane transporter
*28k_302_E09	1.02 ± 0.08	1.15 ± 0.07	1.08 ± 0.09	0.54 ± 0.12	2.15 ± 0.56	2.0E-05	2-oxoisovalerate dehydrogenase, /3-methyl-2-oxobutanoate dehydrogenase
*28k_289_G02	1.06 ± 0.04	1.67 ± 0.11	1.49 ± 0.11	0.33 ± 0.10	5.14 ± 1.25	3.95E-07	ATNUDT21 (Arabidopsis thaliana Nudix hydrolase homolog 21)
*28k_148_D10	1.01 ± 0.03	1.27 ± 0.14	1.28 ± 0.18	0.53 ± 0.08	2.37 ± 0.40	1.0E-05	ring finger protein, putative
*28k_215_A08	1.07 ± 0.08	1.30 ± 0.07	1.30 ± 0.17	0.40 ± 0.04	3.25 ± 0.34	1.24E-08	basic helix-loop-helix (bHLH) family protein
28k_275_A02	1.14 ± 0.13	1.19 ± 0.09	1.20 ± 0.12	0.53 ± 0.08	2.25 ± 0.38	1.22E-06	similar to loricrin-related (TAIR:AT5G64550.1)
*28k_213_B03	1.22 ± 0.09	1.11 ± 0.09	1.00 ± 0.06	0.22 ± 0.04	4.94 ± 0.91	6.33E-08	CYP71B35 (cytochrome P450, family 71, subfamily B, polypeptide 35)
*28k_235_D07	1.26 ± 0.10	1.24 ± 0.10	1.01 ± 0.14	0.18 ± 0.02	6.72 ± 0.80	1.97E-07	CYP71B36 (cytochrome P450, family 71,

							subfamily B, polypeptide 36); oxygen binding
*28k_227_D09	1.16 ± 0.03	1.17 ± 0.05	1.03 ± 0.07	0.38 ± 0.05	3.05 ± 0.57	1.15E-08	CYP71B26 (cytochrome P450, family 71, subfamily B, polypeptide 26)
*28k_114_E06	1.13 ± 0.12	1.11 ± 0.10	1.15 ± 0.15	0.38 ± 0.13	2.94 ± 1.26	2.17E-06	GATL1/GLZ1/PARVUS (Galacturonosyltransferase-Like 1)
28k_282_D11	1.11 ± 0.05	1.38 ± 0.07	1.00 ± 0.09	0.33 ± 0.04	4.11 ± 0.42	2.86E-08	XH/XS domain-containing protein / XS zinc finger domain-containing protein
*28k_077_C05	1.10 ± 0.12	1.26 ± 0.17	1.02 ± 0.13	0.40 ± 0.07	3.15 ± 0.59	4.3E-04	heavy-metal-associated domain-containing protein
*28k_068_H08	1.10 ± 0.05	1.19 ± 0.24	0.95 ± 0.23	0.34 ± 0.12	3.56 ± 1.90	2.10E-06	ATK3 (Arabidopsis Thaliana Kinesin 3)
28k_191_A09	1.07 ± 0.05	1.19 ± 0.07	1.02 ± 0.09	0.42 ± 0.10	2.87 ± 0.71	4.0E-05	similar to unknown protein (TAIR:AT5G18130.1)
*28k_301_H01	1.03 ± 0.04	1.19 ± 0.04	0.91 ± 0.08	0.43 ± 0.08	2.76 ± 0.48	4.29E-06	ATUBC2 (UBIQUITIN-CONJUGATING ENZYME 2); ubiquitin-protein ligase
*28k_209_F09	1.01 ± 0.10	1.17 ± 0.08	0.98 ± 0.09	0.52 ± 0.09	2.24 ± 0.43	6.0E-05	ALDH7B4 (Aldehyde Dehydrogenase 7B4)
*28k_242_G08	0.98 ± 0.09	1.20 ± 0.07	1.06 ± 0.23	0.36 ± 0.05	3.33 ± 0.51	7.6E-04	ALDH2B7 (Aldehyde dehydrogenase 2B7)
*28k_302_B01	0.94 ± 0.10	1.16 ± 0.11	1.08 ± 0.09	0.38 ± 0.06	3.04 ± 0.58	4.24E-06	COL4 (CONSTANS-like 4)
*28k_280_A02	0.93 ± 0.10	1.19 ± 0.09	0.97 ± 0.07	0.39 ± 0.07	3.03 ± 0.67	4.0E-05	heavy-metal-associated domain-containing protein
*28k_211_B11	0.90 ± 0.03	1.19 ± 0.08	1.05 ± 0.05	0.43 ± 0.11	2.76 ± 0.74	1.3E-04	SOX (SULFITE OXIDASE)
28k_169_A02	0.97 ± 0.03	1.12 ± 0.01	0.98 ± 0.09	0.27 ± 0.02	4.15 ± 0.39	4.34E-10	NAC domain protein
*28k_234_E12	0.92 ± 0.07	1.18 ± 0.06	0.98 ± 0.14	0.23 ± 0.06	5.09 ± 0.93	2.32E-06	elastin like protein
*28k_189_E06	0.95 ± 0.06	1.11 ± 0.06	1.00 ± 0.04	0.43 ± 0.04	2.58 ± 0.31	5.08E-07	EIN4 (ETHYLENE INSENSITIVE 4)
*28k_222_B12	0.95 ± 0.12	1.09 ± 0.09	1.01 ± 0.10	0.39 ± 0.07	2.80 ± 0.72	1.8E-04	SOX (SULFITE OXIDASE)
28k_166_F10	0.98 ± 0.04	1.12 ± 0.07	1.00 ± 0.07	0.47 ± 0.05	2.39 ± 0.40	8.35E-08	ERD15 (early responsive to dehydration 15)
*28k_015_E02	1.02 ± 0.20	1.11 ± 0.07	1.02 ± 0.10	0.34 ± 0.12	3.25 ± 2.11	4.3E-04	ADH1 (ALCOHOL DEHYDROGENASE 1)

*28k_205_A01	1.03 ± 0.06	1.11 ± 0.04	1.04 ± 0.06	0.43 ± 0.08	2.57 ± 0.69	8.39E-07	CSN6A (COP9 Signalosome Subunit 6A)
28k_209_G05	0.89 ± 0.04	1.09 ± 0.09	1.05 ± 0.07	0.40 ± 0.11	2.73 ± 1.29	4.4E-04	lateral organ boundaries (LOB) domain family
*28k_178_G09	0.90 ± 0.02	1.07 ± 0.13	1.07 ± 0.05	0.51 ± 0.05	2.10 ± 0.29	2.44E-06	metal ion binding
*28k_102_B04	0.86 ± 0.05	1.10 ± 0.13	1.04 ± 0.10	0.48 ± 0.09	2.28 ± 0.49	3.0E-05	metal ion binding
*28k_229_B03	1.01 ± 0.15	1.03 ± 0.16	1.16 ± 0.28	0.40 ± 0.09	2.54 ± 0.65	1.8E-04	EDA38 (embryo sac development arrest 38); selenium binding
*28k_157_H04	0.98 ± 0.07	1.09 ± 0.04	1.17 ± 0.12	0.45 ± 0.08	2.40 ± 0.59	6.37E-07	PDE135 (PIGMENT DEFECTIVE EMBRYO 135); transmembrane transporter
*28k_260_E01	0.93 ± 0.09	1.02 ± 0.07	1.11 ± 0.12	0.43 ± 0.06	2.35 ± 0.41	2.0E-05	PDE135 (PIGMENT DEFECTIVE EMBRYO 135); transmembrane transporter
*28k_235_D02	0.79 ± 0.03	1.09 ± 0.06	0.65 ± 0.04	0.26 ± 0.09	4.18 ± 1.90	2.64E-06	HSP17.6II (17.6 KDA CLASS II HEAT SHOCK PROTEIN)
*28k_222_G04	0.83 ± 0.07	1.06 ± 0.05	0.71 ± 0.06	0.30 ± 0.05	3.48 ± 0.71	1.74E-06	glycosyl hydrolase family 17
*28k_036_E03	0.74 ± 0.03	1.08 ± 0.30	0.69 ± 0.05	0.30 ± 0.05	3.55 ± 1.03	1.13E-06	transferase family
*28k_188_C07	0.88 ± 0.10	1.11 ± 0.05	0.67 ± 0.08	0.34 ± 0.10	3.21 ± 1.41	7.7E-04	ALDH3I1 (Aldehyde dehydrogenase 3I1) (NAD)
*28k_180_F06	0.84 ± 0.04	0.91 ± 0.07	0.69 ± 0.04	0.39 ± 0.03	2.30 ± 0.29	1.16E-06	glucosamine/galactosamine-6-phosphate isomerase family
*28k_212_D08	1.03 ± 0.05	1.07 ± 0.05	0.94 ± 0.08	0.34 ± 0.05	3.15 ± 0.58	4.96E-08	pfkB-type carbohydrate kinase family protein
*28k_072_C02	0.97 ± 0.07	1.01 ± 0.06	0.90 ± 0.09	0.28 ± 0.08	3.66 ± 1.46	5.06E-07	DORMANCY-ASSOCIATED PROTEIN 1
*28k_181_C02	0.99 ± 0.09	1.00 ± 0.05	0.85 ± 0.06	0.29 ± 0.09	3.44 ± 1.45	1.31E-06	dormancy-associated protein -related
*28k_176_H07	1.03 ± 0.05	1.02 ± 0.09	0.89 ± 0.05	0.42 ± 0.08	2.42 ± 0.50	4.43E-07	AtGRF8 (Growth-Regulating Factor 8)
28k_175_A04	1.15 ± 0.07	1.06 ± 0.07	0.81 ± 0.04	0.31 ± 0.07	3.45 ± 0.93	1.53E-07	similar to unknown protein (TAIR:AT3G15450.1)
28k_162_E12	1.13 ± 0.05	1.14 ± 0.07	0.79 ± 0.04	0.34 ± 0.05	3.32 ± 0.53	5.46E-08	similar to unknown protein (TAIR:AT3G15450.1)

	*28k_208_F01	1.02 ± 0.12	1.04 ± 0.20	0.89 ± 0.10	0.51 ± 0.13	2.05 ± 0.96	3.4E-04	SUS4; sucrose synthase
	*28k_282_C04	0.78 ± 0.09	1.08 ± 0.08	0.85 ± 0.19	0.22 ± 0.08	4.87 ± 2.12	3.0E-05	CYP714A1 (cytochrome P450, family 714, subfamily A, polypeptide 1)
	*28k_213_G04	0.85 ± 0.05	1.09 ± 0.06	0.91 ± 0.08	0.29 ± 0.13	3.79 ± 2.27	8.34E-07	trypsin and protease inhibitor family protein
	*28k_050_A12	0.88 ± 0.22	1.09 ± 0.18	0.91 ± 0.09	0.40 ± 0.07	2.76 ± 0.77	4.88E-06	putative 21 kDa trypsin inhibitor
	28k_138_E03	0.87 ± 0.06	1.14 ± 0.16	0.90 ± 0.11	0.40 ± 0.14	2.88 ± 1.10	6.0E-04	unnamed protein product
	28k_168_C02	0.87 ± 0.04	1.05 ± 0.05	0.87 ± 0.02	0.24 ± 0.04	4.37 ± 0.90	5.0E-05	fiber protein Fb28
	*28k_229_E09	0.89 ± 0.08	1.02 ± 0.04	0.93 ± 0.12	0.24 ± 0.05	4.34 ± 1.55	2.57E-08	nodulin MtN21 family
	*28k_120_A04	0.90 ± 0.09	1.05 ± 0.08	0.97 ± 0.10	0.44 ± 0.16	2.38 ± 1.18	2.4E-04	AGP31 (Arabinogalactan-Protein 31)
	28k_123_C04	0.92 ± 0.08	1.02 ± 0.10	0.98 ± 0.04	0.49 ± 0.05	2.09 ± 0.28	3.56E-06	octicosapeptide/Phox/Bem1p (PB1) domain-containing protein
	*28k_089_A12	0.90 ± 0.08	1.09 ± 0.10	0.80 ± 0.04	0.30 ± 0.08	3.57 ± 1.16	2.3E-04	similar to EMB1879 (EMBRYO DEFECTIVE 1879) (TAIR:AT5G49820)
	*28k_170_E09	0.93 ± 0.05	1.09 ± 0.08	0.85 ± 0.07	0.37 ± 0.07	2.96 ± 0.65	2.79E-06	ATNADP-ME2 (NADP-MALIC ENZYME 2)
	28k_293_F03	0.94 ± 0.06	1.07 ± 0.08	0.77 ± 0.10	0.31 ± 0.07	3.43 ± 0.78	2.0E-05	Receptor activity modifying protein 1
	*28k_172_A01	0.96 ± 0.18	1.04 ± 0.06	0.88 ± 0.12	0.50 ± 0.12	2.06 ± 0.58	1.0E-05	aldo/keto reductase family
	*28k_245_C09	0.86 ± 0.10	1.08 ± 0.09	0.83 ± 0.08	0.38 ± 0.05	2.85 ± 0.44	2.4E-04	universal stress protein (USP) family protein
	*28k_115_D08	0.88 ± 0.06	0.98 ± 0.07	0.85 ± 0.08	0.32 ± 0.09	3.09 ± 1.40	3.09E-06	ATP-binding region, ATPase-like domain-containing protein
	*28k_027_F02	0.89 ± 0.08	0.98 ± 0.09	0.82 ± 0.10	0.32 ± 0.10	3.02 ± 1.68	1.8E-04	auxin-repressed protein
	*28k_073_G09	0.87 ± 0.06	0.96 ± 0.11	0.88 ± 0.07	0.48 ± 0.05	2.01 ± 0.35	1.0E-05	BAM1/BMY7/TR-BAMY (beta-amylase 1)
	*28k_285_A12	0.81 ± 0.09	1.21 ± 0.17	0.92 ± 0.18	0.47 ± 0.09	2.59 ± 0.78	6.2E-04	GCL1 (GCR2-LIKE 1); catalytic
**Class b**	**Array ID.**	**May / CR**	**Aug / CR**	**Nov / CR**	**Feb / CR**	**Feb / Aug**	**P**	**Annotation**

*28k_094_G01	0.82 ± 0.46	0.83 ± 0.22	0.82 ± 0.63	2.44 ± 1.69	2.94 ± 1.74	1.1E-04	terpene synthase/cyclase family protein
*28k_063_A12	0.94 ± 0.28	0.86 ± 0.20	0.89 ± 0.29	1.99 ± 0.89	2.31 ± 0.93	7.0E-04	(+)-delta-cadinene synthase
*28k_231_E03	1.15 ± 0.08	0.91 ± 0.21	0.96 ± 0.11	2.69 ± 0.76	2.96 ± 0.83	2.3E-04	CYCP4; cyclin-dependent protein kinase
*28k_006_F11	1.05 ± 0.10	0.87 ± 0.14	0.93 ± 0.12	1.94 ± 0.43	2.23 ± 0.48	6.09E-06	Catalase
28k_203_C07	0.97 ± 0.10	0.96 ± 0.32	0.98 ± 0.15	2.53 ± 0.53	2.64 ± 0.59	2.0E-05	hypothetical protein
28k_223_E06	1.01 ± 0.10	0.96 ± 0.22	1.01 ± 0.14	2.25 ± 0.39	2.34 ± 0.51	6.61E-06	hypothetical protein
*28k_116_D07	0.96 ± 0.14	0.98 ± 0.09	1.08 ± 0.28	2.06 ± 0.33	2.10 ± 0.55	1.0E-04	RDR6 (RNA-dependent RNA polymerase 6)
*28k_163_D04	0.94 ± 0.16	0.96 ± 0.25	1.03 ± 0.15	2.15 ± 0.47	2.24 ± 0.53	6.20E-06	predicted protein
*28k_300_H11	0.96 ± 0.17	0.96 ± 0.31	1.05 ± 0.13	1.94 ± 0.19	2.02 ± 0.29	9.0E-05	Protein-tyrosine phosphatase-like member B
28k_200_D03	1.02 ± 0.09	0.92 ± 0.14	1.03 ± 0.07	2.21 ± 0.47	2.40 ± 0.50	2.0E-05	mitotic checkpoint family protein
*28k_201_A09	0.98 ± 0.08	0.92 ± 0.18	1.06 ± 0.05	2.13 ± 0.60	2.32 ± 0.68	5.0E-05	AGD8 (ARF-GAP DOMAIN 8); DNA binding
*28k_201_F03	1.04 ± 0.09	0.96 ± 0.19	1.07 ± 0.12	2.06 ± 0.53	2.15 ± 0.57	3.47E-06	Serine/Threonine Protein Kinase 1
*28k_100_H07	0.97 ± 0.18	0.76 ± 0.10	1.05 ± 0.13	1.94 ± 0.28	2.55 ± 0.34	4.29E-06	DCL3 (DICER-LIKE 3)
*28k_047_E10	0.93 ± 0.11	0.86 ± 0.19	1.00 ± 0.18	1.83 ± 0.19	2.13 ± 0.35	4.21.0E-04	CMT3 (chromomethylase 3)
*28k_125_H05	1.05 ± 0.13	0.93 ± 0.16	1.19 ± 0.16	2.30 ± 0.37	2.47 ± 0.43	9.63E-06	leucine-rich repeat transmembrane protein kinase
*28k_302_C07	1.16 ± 0.14	0.91 ± 0.16	1.16 ± 0.08	2.10 ± 0.42	2.31 ± 0.47	4.01E-06	leucine-rich repeat transmembrane protein kinase
28k_256_E07	1.19 ± 0.19	0.98 ± 0.21	1.39 ± 0.35	2.26 ± 1.09	2.31 ± 1.11	7.0E-05	dehydration-responsive family protein
28k_258_A02	1.24 ± 0.17	0.91 ± 0.25	1.36 ± 0.31	2.06 ± 1.18	2.26 ± 1.24	1.0E-04	dehydration-responsive family protein
*28k_041_D07	1.01 ± 0.12	0.83 ± 0.20	1.33 ± 0.19	2.19 ± 0.77	2.64 ± 0.82	4.5E-04	chloroplast-encoded ribosomal protein S18
*28k_284_F07	1.05 ± 0.06	0.86 ± 0.15	1.22 ± 0.09	1.84 ± 0.45	2.14 ± 0.49	9.97E-06	gibberellin-responsive protein
*28k_202_G02	1.04 ± 0.16	0.73 ± 0.19	1.29 ± 0.22	1.81 ± 0.56	2.48 ± 0.70	5.5E-04	30S chloroplast ribosomal protein S14
*28k_248_D02	0.87 ± 0.08	0.74 ± 0.06	1.23 ± 0.09	1.86 ± 0.58	2.51 ± 0.59	5.9E-04	ATLP-1 (Arabidopsis thaumatin-like protein 1)
*28k_297_C12	0.93 ± 0.12	0.76 ± 0.10	1.11 ± 0.26	1.61 ± 0.43	2.12 ± 0.46	4.0E-04	nucleic acid binding protein, putative

*28k_163_G11	1.08 ± 0.11	0.67 ± 0.08	1.55 ± 0.12	1.51 ± 0.43	2.25 ± 0.48	9.1E-04	SGS3 (Suppressor Of Gene Silencing 3)
28k_119_A01	1.05 ± 0.13	0.56 ± 0.06	1.50 ± 0.40	1.28 ± 0.29	2.29 ± 0.35	1.5E-04	hypothetical protein
28k_159_B07	0.87 ± 0.19	0.58 ± 0.10	0.95 ± 0.13	1.69 ± 0.58	2.91 ± 0.64	8.9E-04	No hits
*28k_250_E10	0.87 ± 0.10	0.53 ± 0.05	0.91 ± 0.12	1.37 ± 0.22	2.58 ± 0.27	3.2E-04	DRM2 (Domains Rearranged Methyltransferase 2)
*28k_191_A06	0.90 ± 0.08	0.64 ± 0.15	0.87 ± 0.12	1.32 ± 0.26	2.06 ± 0.41	7.10E-06	RDR1 (RNA-Dependent RNA Polymerase 1)
*28k_101_G08	1.00 ± 0.11	0.66 ± 0.14	1.11 ± 0.30	1.47 ± 0.46	2.23 ± 0.59	6.3E-04	unnamed protein product
28k_259_D05	0.81 ± 0.14	0.54 ± 0.17	1.21 ± 0.07	1.31 ± 0.30	2.43 ± 0.64	1.0E-04	No hits
*28k_251_F10	1.00 ± 0.08	0.45 ± 0.04	1.04 ± 0.16	1.19 ± 0.23	2.64 ± 0.29	1.8E-04	Ocs element-binding factor, putative
*28k_060_H07	0.91 ± 0.13	0.47 ± 0.08	0.97 ± 0.12	1.10 ± 0.20	2.34 ± 0.43	1.1E-04	heavy-metal-associated domain-containing protein
28k_136_F03	1.06 ± 0.12	0.43 ± 0.08	0.87 ± 0.12	1.07 ± 0.11	2.49 ± 0.44	5.5E-04	putative uncharacterized protein At1g72240

[a]Upregulation scored by >2.0-fold increase with false discovery rate–corrected P values < 0.001.

***** Indicates 176 genes with GO annotations.
