# Supplementary material for: A Potential Role for CHH DNA Methylation in Cotton Fiber Growth Patterns
Source: PLoS One. 2013 Apr 12;8(4):e60547. doi: 10.1371/journal.pone.0060547 (PMC3625195; doi:10.1371/journal.pone.0060547)
Supplement: Table S3 — Gene ontology (GO) end-terms of significantly upregulated genes. (PDF) [file pone.0060547.s007.pdf]

Table S3. Gene Ontology (GO) End-Terms of Significantly Upregulated Genes[a]

Serial no.	GO ID	GO term	No. upregulated genes[b]	No. genes in each GO ID	Q value[c]
1	GO:0006306	DNA methylation	5	17	2.49E-06
2	GO:0043617	cellular response to sucrose starvation	2	5	0.0008
3	GO:0030702	chromatin silencing at centromere	2	5	0.0008
4	GO:0031060	regulation of histone methylation	2	5	0.0008
5	GO:0009755	hormone-mediated signaling	15	521	0.0011
6	GO:0009693	ethylene biosynthesis	3	18	0.0015
7	GO:0006529	asparagine biosynthesis	2	8	0.0029
8	GO:0006572	tyrosine catabolic process	2	8	0.0029
9	GO:0009646	response to absence of light	2	9	0.0041
10	GO:0009269	response to desiccation	2	10	0.0050
11	GO:0032270	positive regulation of cellular protein metabolic process	2	10	0.0050
12	GO:0009739	response to gibberellin	4	52	0.0053
13	GO:0009835	ripening	3	29	0.0065
14	GO:0016246	RNA interference	3	29	0.0065

[a]Of 28,178 cotton UniESTs printed on the microarray, 17,430 were annotated in GO (cut-off E-value = $1e^{-5}$) and used for GOEAST analysis.

[b] Number of genes preferentially upregulated in either summer or winter samples.

[c] False discovery rate–corrected P value.
