# Supplementary material for: A Potential Role for CHH DNA Methylation in Cotton Fiber Growth Patterns
Source: PLoS One. 2013 Apr 12;8(4):e60547. doi: 10.1371/journal.pone.0060547 (PMC3625195; doi:10.1371/journal.pone.0060547)
Supplement: Table S4 — Detailed sequence information for genes represented in GO ID: 0006306. (PDF) [file pone.0060547.s008.pdf]

Table S4. Detailed Sequence Information for Genes Represented in GO ID: 0006306

Array ID	Enzyme	Best hit in NCBI[a]	cDNA length (bp)[b]	ORF length (bp)	Predicted protein MW (kDa)	Amino acid identity	Conserved domain[c]	E value
28k_250_E10	DRM1/2	NP_196966	2261 / 2183	1911	70.85	55%	cd00315	0
28k_047_E10	CMT3	NP_177315	3151 / 2764	2706	101.47	58%	COG0270	0
28k_279_B03	DCL3	NP_189978	5634 / 4744	4968	185.02	51%	cd00593	0
28k_303_B02	ROS1	NP_181190	5492 / 4420	5277	196.80	69%	cd00056	0
28k_222_E11	DME	NP_001078527	6779 / 6475	6189	230.78	64%	cd00056	0

[a]Closest *Arabidopsis thaliana* homolog exhibiting the lowest BLASTX E value compared to the cotton enzyme.

[b]cDNA length of putative cotton gene/cDNA length of best matched *A. thaliana* gene.

[c]The putative open reading frame of each cDNA was translated into amino acid sequence, and conserved domains were searched using BLASTP. cd00315, cytosine-C5-specific DNA methylases; COG0270, site-specific DNA methylase; cd00593, RIBOc, ribonuclease III C terminal domain; cd00056, endonuclease III.
