# Supplementary material for: A Potential Role for CHH DNA Methylation in Cotton Fiber Growth Patterns
Source: PLoS One. 2013 Apr 12;8(4):e60547. doi: 10.1371/journal.pone.0060547 (PMC3625195; doi:10.1371/journal.pone.0060547)
Supplement: Table S5 — Relative expression of 20 Gossypium hirsutum housekeeping genes based on microarray and QRT-PCR analysis. (PDF) [file pone.0060547.s009.pdf]

Table S5. Relative Expression of 20 *Gossypium hirsutum* Housekeeping Genes Based on Microarray and QRT-PCR Analysis

Array ID	Relative expression ratios[a] May	Aug.	Nov.	Feb.	Annotation[b]
28k_212_F04	0.94 ± 0.10	0.93 ± 0.11	0.91 ± 0.05	0.90 ± 0.14	*UBQ7*
	0.96 ± 0.05	0.99 ± 0.02	1.00 ± 0.12	1.05 ± 0.05	
28k_172_C11	1.02 ± 0.12	1.10 ± 0.06	1.10 ± 0.10	0.99 ± 0.17	*UBQF*
	1.14 ± 0.09	0.97 ± 0.08	0.93 ± 0.05	0.96 ± 0.03	
28k_070_H12	0.96 ± 0.11	0.99 ± 0.09	0.90 ± 0.07	1.02 ± 0.17	*TUA3*
	1.04 ± 0.03	0.95 ± 0.04	0.92 ± 0.04	1.10 ± 0.09	
28k_211_H07	1.08 ± 0.07	1.07 ± 0.07	1.06 ± 0.09	1.13 ± 0.14	*TUA8*
	1.02 ± 0.07	0.98 ± 0.07	0.97 ± 0.08	1.03 ± 0.08	
28k_284_E12	1.00 ± 0.05	0.97 ± 0.08	0.95 ± 0.07	0.93 ± 0.09	*TUB2*
	1.05 ± 0.13	0.99 ± 0.02	0.96 ± 0.05	1.00 ± 0.05	
28k_285_C10	1.01 ± 0.02	1.01 ± 0.05	0.99 ± 0.04	1.12 ± 0.24	*TUB10*
	1.00 ± 0.03	1.02 ± 0.12	0.99 ± 0.11	0.99 ± 0.11	
28k_242_B06	1.01 ± 0.10	0.93 ± 0.13	0.96 ± 0.09	1.03 ± 0.14	*TUB18*
	1.07 ± 0.08	0.97 ± 0.06	1.02 ± 0.04	0.94 ± 0.02	
28k_099_G03	0.95 ± 0.02	0.94 ± 0.06	0.95 ± 0.07	1.03 ± 0.14	*RPL8C*
	1.04 ± 0.14	0.94 ± 0.07	1.04 ± 0.12	0.98 ± 0.09	
28k_170_E03	0.92 ± 0.05	0.93 ± 0.03	0.97 ± 0.07	0.98 ± 0.14	*RPL19B*
	1.12 ± 0.05	0.91 ± 0.04	0.94 ± 0.02	1.04 ± 0.06	
28k_220_B09	0.99 ± 0.06	0.92 ± 0.09	1.02 ± 0.07	1.02 ± 0.10	*RPS4A*
	0.97 ± 0.05	0.93 ± 0.06	1.05 ± 0.04	1.05 ± 0.03	
28k_211_B03	0.95 ± 0.11	1.04 ± 0.10	1.02 ± 0.08	1.00 ± 0.06	*RPSA*
	0.95 ± 0.04	1.01 ± 0.03	1.11 ± 0.08	0.94 ± 0.02	
28k_170_A12	0.91 ± 0.06	0.91 ± 0.08	1.06 ± 0.09	1.01 ± 0.08	*RPS9C*
	0.93 ± 0.10	1.06 ± 0.04	1.02 ± 0.06	0.98 ± 0.02	
28k_121_D07	1.07 ± 0.16	0.94 ± 0.10	1.04 ± 0.12	0.93 ± 0.15	*eIF2A*
	1.05 ± 0.12	0.95 ± 0.16	1.02 ± 0.09	0.98 ± 0.10	
28k_284_D01	1.08 ± 0.11	1.07 ± 0.11	1.05 ± 0.11	0.99 ± 0.17	*eIF2B*
	1.05 ± 0.10	1.00 ± 0.09	1.01 ± 0.09	0.95 ± 0.09	
28k_273_D08	1.12 ± 0.04	1.15 ± 0.12	1.12 ± 0.05	1.07 ± 0.19	*eIF3B*
	1.07 ± 0.16	1.03 ± 0.15	0.94 ± 0.02	0.96 ± 0.13	
28k_088_G09	1.07 ± 0.16	0.95 ± 0.06	1.05 ± 0.10	1.01 ± 0.07	*eIF4A*
	0.95 ± 0.06	1.04 ± 0.04	0.95 ± 0.08	1.06 ± 0.02	
28k_184_A01	0.92 ± 0.07	1.10 ± 0.18	0.92 ± 0.08	0.95 ± 0.10	*GAPDH*
	0.97 ± 0.10	1.00 ± 0.05	0.94 ± 0.08	1.08 ± 0.03	

28k_207_F09	0.92 ± 0.07	0.95 ± 0.05	0.93 ± 0.08	1.02 ± 0.16	*COX*
	0.96 ± 0.12	1.05 ± 0.08	1.04 ± 0.04	0.94 ± 0.04	
28k_121_A02	1.02 ± 0.10	0.96 ± 0.06	1.08 ± 0.06	0.95 ± 0.12	*ACT7*
	0.95 ± 0.05	1.01 ± 0.10	0.92 ± 0.01	1.12 ± 0.09	
28k_282_A09	0.96 ± 0.07	1.00 ± 0.05	1.02 ± 0.07	1.04 ± 0.11	*FBA*
	1.04 ± 0.11	0.97 ± 0.08	1.01 ± 0.04	0.99 ± 0.02	

[a]Relative expression ratios of May, August, November, and February compared to CR based on microarray (top) and QRT-PCR (bottom) analysis.

[b]*UBQF*, ubiquitin family; *TUA*, tubulin alpha; *TUB*, tubulin beta; *RPL*, 60S ribosomal protein; *RPS*, 40S ribosomal protein; *eIF*, eukaryotic translation initiation factor; *GAPDH*, glyceraldehyde-3-phosphate dehydrogenase; *COX*, cytochrome c oxidase subunit; *ACT*, actin; *FBA*, fructose-bisphosphate aldolase.
