# Supplementary material for: A Potential Role for CHH DNA Methylation in Cotton Fiber Growth Patterns
Source: PLoS One. 2013 Apr 12;8(4):e60547. doi: 10.1371/journal.pone.0060547 (PMC3625195; doi:10.1371/journal.pone.0060547)
Supplement: Table S6 — Primers used in the current work. (PDF) [file pone.0060547.s010.pdf]

**Table S6.** Primers Used in the Current Work

| Gene | Sequence | Amplified fragment length (bp) |
|---|---|---|
| | **QRT-PCR analysis** | |
| *CMT3* | 5’ - TTACCAAGTCCGAATGGGAATGAT | 421 |
| | 5’ - TCATCCGTGTTGAGTTGAAGTGG | |
| *DRM1/2* | 5’ - CATCCCTCTTATCGCTGCCTTCC | 352 |
| | 5’ - AAGCAGACCTTACTTCTCGTGGG | |
| *DCL3* | 5’ - TTGGTAGGAGAGGGGTTTGATAG | 245 |
| | 5’ - ACTGGGGTGGCAGGCGTAG | |
| *ROS1* | 5’ - CAACAGTTACAAGCCGCATCCAC | 334 |
| | 5’ - AAGGTACTTGCACGGGTCGTCAG | |
| *DME* | 5’ - ATGCTGCTTCTATCCCTACTCCA | 126 |
| | 5’ - GATCATCAGGTTCCCGCTTTT | |
| *UBQ7* | 5’ - GAAGGCATTCCACCTGACCAAC | 198 |
| | 5’ - CTTGACCTTCTTCTTCTTGTGCTTG | |
| *UBQF* | 5’ - GCCAGATGCTTCTCAGTTGACTC | 246 |
| | 5’ - GCTGGGATAGAAGTGATTGCTGT | |
| *TUA3* | 5’ - GAGGTTTTCTCCCGAATCGAC | 231 |
| | 5’ - CATTGACTGCGACTGCTGATTT | |
| *TUA8* | 5’ - AGGTGCAGCGAGCTGTTTGT | 295 |
| | 5’ - CCTCACAGGGTGAACCCATC | |
| *TUB2* | 5’ - CTGGTGAGGGAATGGATGAGAT | 282 |
| | 5’ - ACAAAGGAACGAACAGAGCAGAA | |
| *TUB10* | 5’ - ACTGGTGAAGGGATGGATGAAA | 296 |
| | 5’ - CAAGGCTGGAATGCAAACATAA | |
| *TUB18* | 5’ - TGGCAAAATGAGCACTAAGGAA | 383 |
| | 5’ - CCTAATCCTCGTACTCCGCTTC | |
| *RPL8C* | 5’ - CGTCGTCTTCCGTCATCCA | 356 |
| | 5’ - GAGGTTTCTCGGTCCTTCCA | |
| *RPL19B* | 5’ - ATCCACAAGTCCAAGGCTGAG | 347 |
| | 5’ - CACAAACATCTTCCATCTTCCATT | |
| *RPS4A* | 5’ - AAGATGCTGCTGGGCACGAA | 269 |
| | 5’ - TCTATGACATCACCCACAACACAA | |
| *RPSA* | 5’ - ACAAACCCCTGGTGCTCCTA | 132 |
| | 5’ - TTCCATTGTTCGACATAGCTCATA | |
| *RPS9C* | 5’ - ATTAGGGTTGGTAGGCAAGTGG | 262 |

| | | |
|---|---|---|
| | 5’ - CAATGGAGGTGATGGCAAAGT | |
| *eIF2A* | 5’ - GTGAGCGAACGGGATGATAAA | 238 |
| | 5’ - TCCATGAGATGAGGCCAATTG | |
| *eIF2B* | 5’ - TAGGTGTGCTTACTCCTTCCGTC | 261 |
| | 5’ - TGGCAATCTTTGCATTCTTCAA | |
| *eIF3B* | 5’ - GCTTGAAACAATGGCAACTGC | 363 |
| | 5’ - CCATTCGCTTATCCATTTTTCC | |
| *eIF4A* | 5’ - TGGTCGTTCAGGTCGCTTTG | 239 |
| | 5’ - TGCCTTCAGTTTCCACGGTG | |
| *GAPDH* | 5’ - TTGTTGGCGATTCCAGGTCT | 270 |
| | 5’ - TCACTGATTTATGTCCCGTGCA | |
| *COX* | 5’ - GCTCCTGCTGTTGTCAAGTCCT | 196 |
| | 5’ - GATCATCATCGCCATGTCCG | |
| *ACT7* | 5’ - AGAATGAGCAAGGAAATCACAGC | 255 |
| | 5’ - CGAAACCCAAGCCAAACCA | |
| *FBA* | 5’ - CAAAGTGAGGAGGAGGCTACCC | 329 |
| | 5’ - CCCCCAAAATCATCAATACAGAA | |
| *KCS13* | 5’ - AAGCGTTTGAACACTTCTGTATCCA | 287 |
| | 5’ - TGGTCCATCTTTTGGTGTTTTG | |
| *SUR4* | 5’ - TAATGGCGATGCTTGACTTCTA | 150 |
| | 5’ - ATTGTTGACCTTTGGGGGAGT | |
| *ERF6* | 5’ - CCCACCAAAAATCAAGCAGC | 211 |
| | 5’ - ATTCCAGGCTCCAGATTTGTTC | |
| **Bisulfite sequencing analysis** | | |
| *KCS13* | 5' - TTATTGATGAAAGYAAGGGTTTA | 299 |
| | 5' - AATTAATCCCTTTATRTTAAATCA | |
| *SUR4* | 5' - TTTTAATTGTTTGGGGGTGTTT | 366 |
| | 5' - TCCCATTCTATCTTTTRTTRAAA | |
| *ERF6* | 5’ - GGGTGGYGGATTATGTTA | 391 |
| | 5' - TTATTATTRTCATTRATCCCTAART | |
| *TUB3* | 5' - TATAYTTTTYGAATTTTAAAATTT | 362 |
| | 5' - AAARRAAAATACCTTAATTTAACA | |
| *EIF2A* | 5' - TGTATTYGTYAGGAGYAAGGTA | 450 |
| | 5' - TTRATAATTATCCTTCTCCCTRT | |
| **Methylation-sensitive Southern blotting** | | |
| *KCS13 -466 ~ -178* | 5’ - TGTCTGGTTATTCCGTGAGCC | 289 |
| | 5’ - GCAGTCAGGCAGATGAGAGGAT | |
| *SRU4 -1155 ~ -856* | 5’ - GATAAGAGCTTCTAAATAGGTTTATGGTTG | 300 |

| | | |
|---|---|---|
| | 5’ - CAATACTTAATACTTGTTAATGTAGTTCGG | |
| *ERF6 -263 ~ -21* | 5’ - CACCGGGCACAATTTTTCACA | 243 |
| | 5’ - CTCCATAACACAAGGTATTATTTCTGGG | |
| *ERF6 -173 ~ -14* | 5’ - ACCTCCAAAATGTCCCTCACTT | 187 |
| | 5’ - GCACCTCCACACATAGACCAAGA | |
| **Methylation-sensitive PCR** | | |
| *KCS13 -770 ~ -447* | 5’ - GAAGCCTGAGACCAACCGATAT | 324 |
| | 5’ - GCTCACGGAATAACCAGACAAT | |
| *SUR4 -1014 ~ -346* | 5’ - GAGTTTGACTTCGGGGTTGC | 669 |
| | 5’ - CCACCCTACCATCTACTCTGTTTT | |
| *ERF6 -594 ~ -96* | 5’ - CACCGGGCACAATTTTTCACA | 499 |
| | 5’ - CTCCATAACACAAGGTATTATTTCTGGG | |
| *ERF6 -496 ~ -90* | 5’ - GCGTCCAACATTCTACATTCCTAC | 407 |
| | 5’ - TCAGAAGGTGTGTAGCCGAGAG | |
| **RNAi line vector** | | |
| *ROS1 pos* | 5’ - GAGCTGGTGGGATGAATAATGTGCTTGCTC | 549 |
| | 5’ - CCCGGGCAGATGTTCCATTTTCAGTAGCAC | |
| *ROS1 neg* | 5’ - CTCGAGGTGGGATGAATAATGTGCTTGCTC | 549 |
| | 5’ - GTCGACCAGATGTTCCATTTTCAGTAGCAC | |
| *NPTII* | 5’ - GCGATACCGTAAAGCACGAGGAA | 417 |
| | 5’ - TCCTGCCGAGAAAGTATCCATCA | |
