# Supplementary material for: A Potential Role for CHH DNA Methylation in Cotton Fiber Growth Patterns
Source: PLoS One. 2013 Apr 12;8(4):e60547. doi: 10.1371/journal.pone.0060547 (PMC3625195; doi:10.1371/journal.pone.0060547)
Supplement: Table S7 — Statistical analysis of methylation pattern changes in all the cytosine sites present in the upstream regions shown in Figure 3 . (PDF) [file pone.0060547.s011.pdf]

Table S7. Statistical Analysis of Methylation Pattern Changes in All the Cytosine Sites Present in the Upstream Regions Shown in Figure 3.

	**Cytosine Sites No.**	**Feb.**	**May**	**Aug.**	**Nov.**	**Feb. 2011**	**P value#**
**a**	1	0	1	0	0	0	N/A
	2	0	1	2	0	2	0.145
	3	17	16	17	17	17	N/A
	4	16	17	17	17	17	0.310
	5	1	1	0	0	0	0.310
	6	9	7	4	7	10	0.037
	7	0	1	0	1	0	N/A
	8	10	7	5	9	11	0.084
	9	3	5	2	3	5	0.628
	10	1	0	2	1	1	0.545
	11	7	5	3	3	7	0.132
	12	1	1	1	1	3	1.000
	13	9	4	5	4	9	0.163
	14	4	0	0	1	4	0.146
	15	13	10	7	9	13	0.037
	16	7	0	3	3	7	0.132
	17	0	0	0	0	0	N/A
	18	0	0	0	0	1	N/A
	19	16	17	15	16	17	0.545
	20	1	0	0	0	1	0.310
	21	2	2	1	1	2	0.545
	22	0	0	2	1	0	0.145
	23	0	0	0	0	1	N/A
	24	6	8	4	5	7	0.452
	25	0	0	0	0	0	N/A
	26	0	3	0	1	1	N/A
	27	0	2	2	1	0	0.145
	28	1	2	0	0	1	0.310
	29	1	0	0	0	1	0.310
	30	1	2	2	4	2	0.545
	31	0	0	0	0	0	N/A
	32	0	0	0	0	0	N/A
	33	0	0	0	0	1	N/A
	34	16	17	17	17	17	0.310
	35	0	1	0	1	2	N/A

36	17	17	16	17	17	0.310
37	0	0	0	0	0	N/A
38	1	2	1	0	0	1.000
39	0	1	3	2	1	0.070
40	0	0	0	1	0	N/A
41	0	1	0	0	1	N/A
42	16	11	12	15	17	0.072
43	3	4	3	3	3	1.000
44	6	2	5	6	6	0.714
45	3	3	2	1	2	0.628
46	3	0	0	1	2	0.070
47	9	6	6	6	9	0.300
48	2	0	0	1	1	0.145
49	2	3	3	3	3	0.628
50	8	3	0	2	8	0.001
51	1	1	0	1	1	0.310
52	2	7	0	1	5	0.145
53	0	0	0	0	0	N/A
54	2	2	2	0	3	1.000
55	1	0	0	1	1	0.310
56	11	8	5	7	10	0.039
57	0	0	0	0	0	N/A
58	17	17	17	17	16	N/A
59	17	17	16	17	17	0.310
60	0	2	1	1	2	0.310
61	7	5	3	5	6	0.132
62	1	0	0	1	1	0.310
63	1	0	0	1	1	0.310
64	17	17	17	17	17	N/A
65	0	1	0	0	0	N/A
66	1	2	4	2	0	0.146
67	0	0	0	1	0	N/A
68	4	2	1	2	5	0.146
69	1	1	0	1	1	0.310
70	0	0	0	0	0	N/A
71	3	2	4	0	3	0.671
72	0	0	0	0	0	N/A
73	0	0	0	0	1	N/A

	74	0	0	3	3	1	0.070
	75	3	5	5	3	4	0.419
	76	0	0	0	0	0	N/A
	77	0	1	0	0	0	N/A
	78	9	7	5	7	11	0.039
	79	14	11	12	13	14	0.419
	80	0	0	2	1	0	0.145
	81	8	5	2	4	8	0.024
	82	17	15	13	16	17	0.033
	83	11	12	11	13	11	1.000
**b**	1	0	0	0	0	0	N/A
	2	3	3	3	3	3	1.000
	3	3	3	3	4	3	1.000
	4	2	2	2	3	3	1.000
	5	11	7	4	8	10	0.016
	6	3	3	3	2	3	1.000
	7	17	17	16	17	17	0.310
	8	16	16	17	16	15	0.310
	9	3	3	3	3	2	1.000
	10	6	6	2	5	6	0.106
	11	17	17	17	16	17	N/A
	12	4	4	4	5	4	1.000
	13	3	3	3	4	4	1.000
	14	10	9	9	9	10	0.730
	15	5	5	4	5	5	0.697
	16	7	7	6	7	9	0.724
	17	10	7	4	8	11	0.037
	18	9	8	8	9	10	0.732
	19	6	6	5	6	5	0.714
	20	16	17	17	17	17	0.310
	21	8	8	7	8	8	0.730
	22	17	16	16	16	16	0.310
	23	7	7	6	7	8	0.724
	24	8	9	8	9	8	1.000
	25	4	4	4	4	6	1.000
	26	10	5	3	6	11	0.013
	27	12	8	5	9	12	0.016

	28	6	5	5	6	7	0.714
	29	10	8	4	7	11	0.037
	30	3	3	3	3	3	1.000
	31	10	7	4	7	10	0.037
	32	12	9	5	9	12	0.016
	33	8	8	5	8	10	0.290
	34	4	4	3	3	5	0.671
	35	1	1	1	2	4	1.000
	36	4	3	3	4	5	0.671
	37	4	4	5	5	5	0.697
	38	8	6	5	5	9	0.290
	39	2	2	2	2	3	1.000
	40	3	4	4	3	5	0.671
	41	2	3	2	3	3	1.000
**c**	1	1	1	2	2	1	0.545
	2	7	6	6	6	7	0.724
	3	2	1	1	2	2	0.545
	4	4	4	3	5	3	0.671
	5	10	9	9	10	10	0.730
	6	13	14	14	14	14	0.671
	7	11	10	10	10	11	0.724
	8	12	11	12	12	12	1.000
	9	11	11	10	10	12	0.724
	10	5	6	5	6	6	1.000
	11	8	8	7	7	8	0.730
	12	10	7	4	7	9	0.037
	13	6	7	6	7	6	1.000
	14	8	5	2	5	7	0.024
	15	17	17	17	16	17	N/A
	16	11	8	4	8	10	0.016
	17	13	11	8	11	12	0.031
	18	12	13	13	14	13	0.697
	19	17	16	16	16	16	0.310
	20	14	14	13	14	15	0.671
	21	14	15	15	16	14	0.628
	22	17	17	16	15	17	0.310
	23	12	10	10	11	10	0.473

24	0	0	0	0	0	N/A
25	10	10	10	10	11	1.000
26	12	12	12	12	12	1.000
27	16	16	16	17	15	1.000
28	8	7	6	7	7	0.486
29	8	7	6	6	8	0.486
30	1	0	1	2	2	1.000
31	6	6	3	7	6	0.244
32	14	16	14	16	14	1.000
33	15	16	15	16	15	1.000
34	11	10	10	11	12	0.724
35	16	16	17	17	17	0.310
36	12	8	5	9	11	0.016
37	10	9	7	9	9	0.303
38	7	7	6	6	7	0.724
39	17	17	17	17	16	N/A
40	12	12	10	11	10	0.473
41	14	15	14	15	14	1.000

[#]Cytosine sites with P < 0.05 were gray shadowed in the Table.

a, *ERF6* upstream region as shown in Figure 3A; b, *SUR4* upstream region as shown in Figure 3B; c, *KCS13* upstream region as shown in Figure 3C.
