# Supplementary material for: A Potential Role for CHH DNA Methylation in Cotton Fiber Growth Patterns
Source: PLoS One. 2013 Apr 12;8(4):e60547. doi: 10.1371/journal.pone.0060547 (PMC3625195; doi:10.1371/journal.pone.0060547)
Supplement: Table S8 — Digital intensities of methylation-sensitive endonuclease-digested and full-length fragments. (PDF) [file pone.0060547.s012.pdf]

Table S8. Digital Intensities of Methylation-Sensitive Endonuclease-Digested and Full-Length Fragments

Gene	Biological replicate	Fragment (bp)	February		May		August		November	
			Intensity[a]	Ratio[b]	Intensity	Ratio	Intensity	Ratio	Intensity	Ratio
SUR4	1	1611	425		299		249		301	
		330	212	2.00	331	0.90	470	0.53	305	0.99
	2	1611	373		296		155		304	
		330	173	2.16	316	0.94	400	0.39	309	0.98
KCS13	1	1716	1300		896		491		954	
		837	525	2.47	655	1.37	1089	0.45	701	1.36
	2	1716	1181		976		576		988	
		837	506	2.33	674	1.45	1036	0.56	724	1.37
ERF6	1	605	627		397		332		393	
		244	337	1.86	422	0.94	651	0.51	460	0.85
	2	605	670		390		304		425	
		244	339	1.98	479	0.81	689	0.44	496	0.86

[a]Intensity, background-subtracted pixel intensity of each fragment on the X-ray film. Data obtained from two independent Southern blots.

[b]Ratio of methylated to unmethylated DNA calculated by dividing the signal of the full-length sequence by that of the corresponding endonuclease-cleaved fragment.
